# Resolving deep-sea pelagic saccopharyngiform eel mysteries: Identification of *Neocyema* and Monognathidae leptocephali and establishment of a new fish family "Neocyematidae" based on larvae, adults and mitogenomic gene orders

**DOI:** 10.1371/journal.pone.0199982

**Published:** 2018-07-25

**Authors:** Jan Y. Poulsen, Michael J. Miller, Tetsuya Sado, Reinhold Hanel, Katsumi Tsukamoto, Masaki Miya

**Affiliations:** 1 Department of Fish and Shellfish, Greenland Institute of Natural Resources, Kivioq, Nuuk, Greenland; 2 Fish Section, Australian Museum, Sydney NSW, Australia; 3 Department of Marine Science and Resources, Nihon University, Fujisawa, Japan; 4 Natural History Museum and Institute, Chiba, Aoba-cho, Chuo-ku, Chiba, Japan; 5 Thunen-Institute of Fisheries Ecology, Hamburg, Germany; Chang Gung University, TAIWAN

## Abstract

Deep-sea midwater “saccopharyngiform” eels of the families Cyematidae, Monognathidae, Eurypharyngidae and Saccopharyngidae (order Anguilliformes) are extraordinary fishes having major skeletal reductions and modifications compared to the general anguilliform body structure. Little is known about most aspects of the systematics, phylogeny, and ecology of these families, and few of the approximately 30 species described from adult specimens have been matched with their leptotocephalus larvae. Based on mitogenomic sequence data from rare new specimens, we show that the long-speculated-about larval form referred to as “*Leptocephalus holti*”, which was thought to possibly be the larva of the rare orange-colored eels of *Neocyema* (5 known specimens; speculated to belong to the Cyematidae) are actually the larvae of the one-jaw eels of the family Monognathidae. One of the 5 types of *L*. *holti* larvae that were collected in the Pacific is genetically matched with *Monognathus jesperseni*, but multiple species exist based on larval sequence data and the morphology of adult specimens. A rare leptocephalus from the Sargasso Sea, with unique morphological characteristics including many small orange spots on the gut, was found to be the larva of *Neocyema*, which is presently only known from the Atlantic Ocean. We demonstrate that *Neocyema* constitutes a separate family being most closely related to Eurypharyngidae and Saccopharyngidae based on mitogenomic DNA sequences and unique mitochondrial gene orders.

## Introduction

The order Anguilliformes (true eels) and their relatives within the Elopomorpha in the orders Albuliformes (notacanths and bonefishes) and Elopiformes (tarpons and ladyfishes) share the common trait of having a leptocephalus larva, which is unique in a variety of ways compared to other fish larvae [1–6]. Most of the 19 families of anguilliform eels share a basic “eel-like” body form, but the exceptions to that typical pattern are found in the meso- and bathy-pelagic eel families that live in the deep-sea and have no association with benthic habitats [5, 7]. The commonly collected mesopelagic sawtooth eels of the Serrivomeridae and the snipe eels of the Nemichthyidae have greatly elongated bodies and jaws to varying degrees, but except for the longneck eels of the Derichthyidae, the other deep-sea eels have several other unusual morphological features.

**Fig 1:**
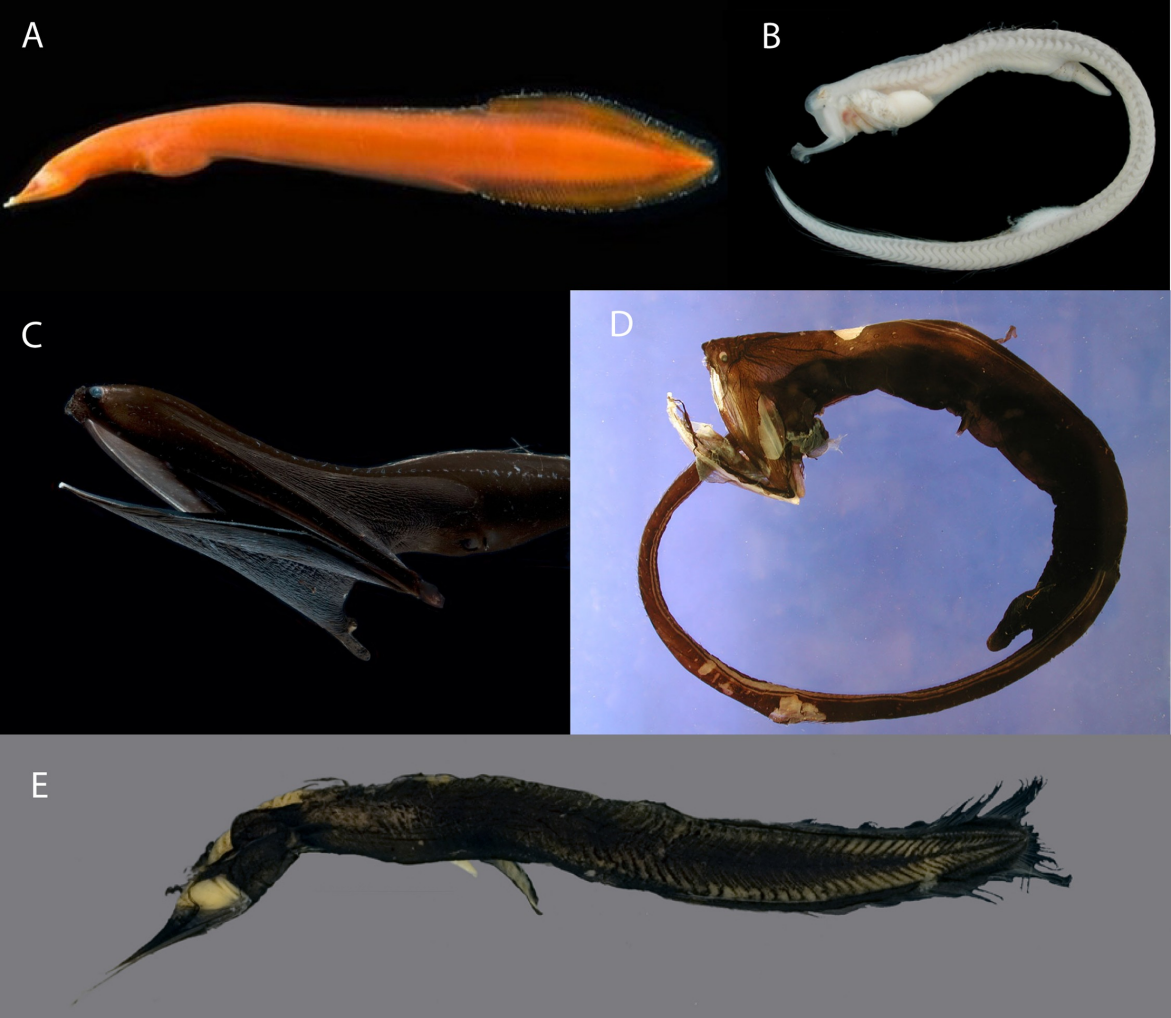
**Five different adult morphotypes of saccopharyngiform fishes (A–E).** A, *Neocyema erythrosoma* (NMS.Z.2010.85.1) 157 mm TL. Photo by D. Shale (MAR-ECO). B, *Monognathus jesperseni* (MCZ 164702) 142 mm SL. Photo by MCZ. C, *Eurypharynx pelecanoides* (LACM 56986–1) 511 mm TL. Photo by D. Shale (MAR-ECO), D, *Saccopharynx ampullaceus* (MCZ 161545) 330 mm TL. Photo by MCZ. E, *Cyema atrum* (MCZ 165935) 130 mm SL. Photo by MCZ. Photos A and C reprinted from http://www.deepseaimages.co.uk and under a CC BY license, with permission from David Shale, original copyright 2009. Photos B, D and E reprinted from http://www.mcz.harvard.edu/Departments/Ichthyology and under a CC BY license, with permission from the Museum of Comparative Zoology, Harvard University, original copyright.

**Fig 2:**
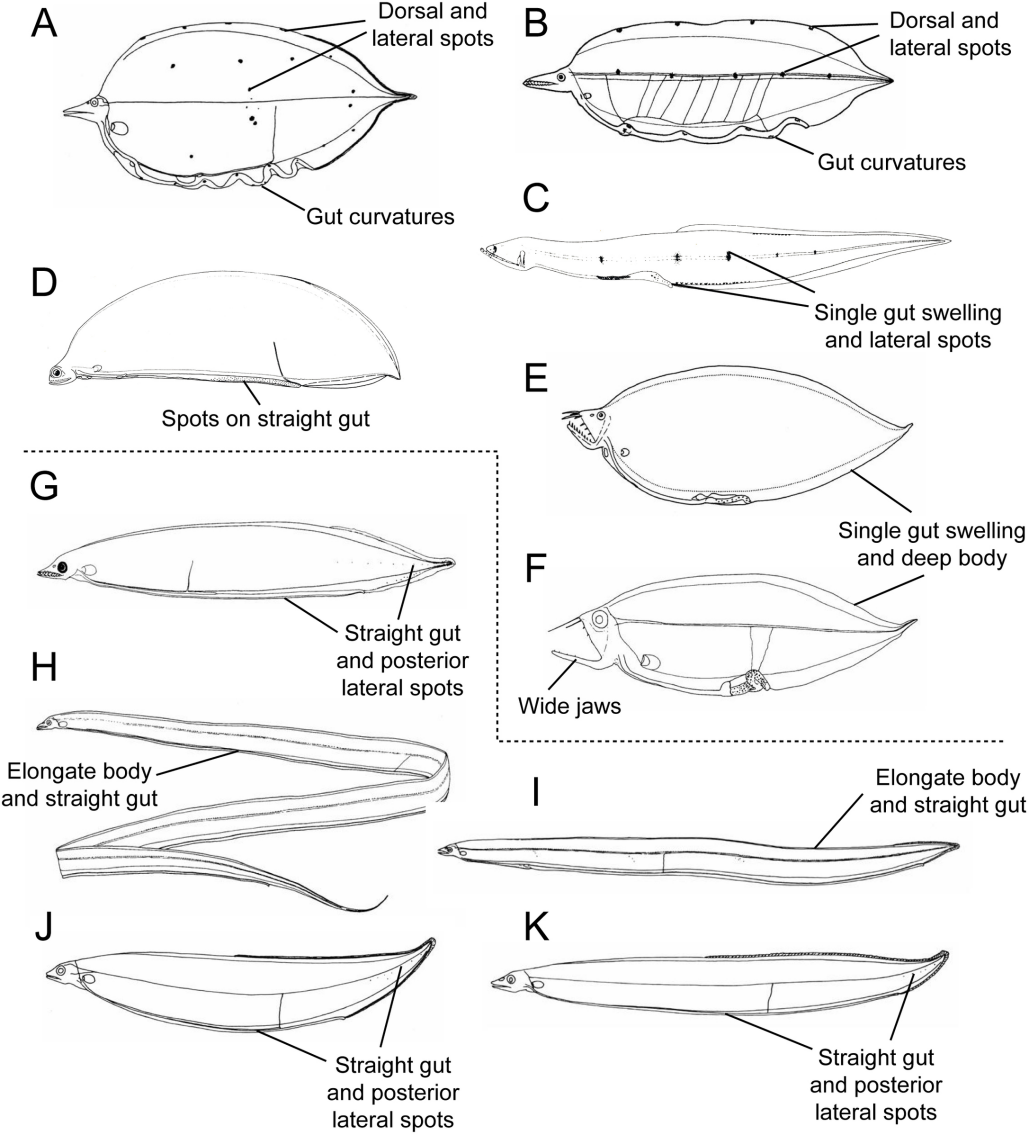
**Line-illustrations of leptocephalus larvae of meso- and bathypelagic anguilliform families (A–K).** A, *Cyema* (Cyematidae). B, “*Leptocephalus holti*”. C, Monognathidae (metamorphic stage). D, Unidentified saccopharyngiform. E, *Saccopharynx* (Saccopharyngidae). F, *Eurypharynx* (Eurypharyngidae). G, *Serrivomer beani* (Serrivomeridae). H, *Nemichthys curvirostris* (Nemichthyidae). I, *Avocettina infans* (Nemichthyidae). J, *Derichthys serpentinus* (Derichthyidae). K, *Nessorhamphus ingolfianus* (Derichthyidae). The dotted line separates the saccopharyngiform larvae from the larvae of other meso- and bathy-pelagic eel families. Illustrations A, C, D, F–K are reproduced or modified from Böhlke [1], B is modified from Smith and Miller [24], and E is modified from Castle [84] with permission under a CC BY license, from the Sears Foundation for Marine Research original copyright 1989, illustrator Mary H. Fuges, Yale University, and the American Society of Ichthyologists and Herpetologists, Lawrence, Kansas, respectively.

The four “saccopharyngiform” families Cyematidae, Eurypharyngidae, Monognathidae and Saccopharyngidae form a separate lineage based on complete mitochondrial (mt) DNA sequences [8] and have a variety of highly derived morphological features. The four families were previously classified as an elopomorph order “Saccopharyngiformes” [2, 9] although the lineage is a derived subclade within the Anguilliformes and ordinal status therefore not appropriate [8]. The Saccopharyngidae (swallowers) and Eurypharyngidae (gulpers) have long-thin tails with luminous organs at the end, greatly extendable guts for holding large prey, and long jaws [10, 11]. The Cyematidae (bob-tail snipe eels) have drastic shortening of the body [9] and the Monognathidae (one-jaws) have reabsorbed upper jaws and poisonous fangs in the adults [11, 12]. *Neocyema*, a genus known from only five specimens [13], also has a shortened body that superficially resembles *Cyema atrum* (Fig 1). The four rare deep-sea families have traditionally been linked phylogenetically because of their incredible modifications and reductions and their deep-bodied leptocephali (Fig 2). However, the biology, evolution, taxonomy and classification of pelagic deep-sea eels within the Elopomorpha constitute major unknowns at present [14], with their intra- and interrelationships being unclear [15]. They represent yet another example of how little is known about the biodiversity of fishes and species divergences, including life stages, within the deep-sea pelagic environment [16, 17].

The leptocephalus larvae of these four families (Figs 1 and 2), which we will refer to here as “saccopharyngiforms”, show some equally unusual characteristics as observed in the adults, and there are some larval types of these and other eels whose adults have remained unknown [18–20]. All of their leptocephali have at least relatively deep bodies compared to the larvae of other mesopelagic eel families (Fig 2, Serrivomeridae, Nemichthyidae and Derichthyidae) as well as most other types of leptocephali [1, 5, 21]. Although no larvae have been attributed to the Monognathidae, the larvae of both Eurypharyngidae and Saccopharyngidae have been identified and they have widely opening mouths with long lower jaws and pigmented swellings at the ends of their short guts, which are unique among all types of leptocephali (Fig 2E and 2F) [22]. Another unknown type of larva was also attributed to being a saccopharyngiform, which had a deep body, a long lower jaw and a straight gut with spots was also reported (Fig 2D) [19, 23]. The larvae of *C*. *atrum* have an exceptionally deep body, a long, pointed snout, gut curvatures, randomly positioned lateral pigment spots, and dorsal pigment spots (Fig 2A) [18]. Another type of leptocephali, referred to as “*Leptocephalus holti*” because its adult species has remained unknown, share the features of a pointed snout, gut curvatures, and dorsal spots, so it has been thought to possibly be a type of cyematid larvae (Fig 2B) [9, 24].

Schmidt [25] described *L*. *holti* from the northeastern Atlantic Ocean in 1909 and it was not reported again until 1974 where Raju [26] found another specimen reminiscent of *L*. *holti* from the South-Central Pacific. This type of leptocephalus has subsequently been collected in a variety of locations in the Atlantic and Pacific [24, 27–29]. It appears to be consistently present in the Sargasso Sea [24, 30], but is always rare in comparison to the abundance of other types of leptocephali [28, 30, 31], and it has not been detected in surveys for leptocephali in the Indonesian Seas [32] or the western Indian Ocean [31]. Smith & Miller [24] showed that there are at least three different morphological types of *L*. *holti* leptocephali representing several different species that included specimens with both lateral and dorsal spots, only dorsal spots, or no spots except on the gut.

Because of its similarities to the leptocephali of *C*. *atrum*, *L*. *holti* has been thought to possibly be the larvae of *Neocyema*, which have also been considered to possibly belong to the Cyematidae [18, 24]. The adults of *Neocyema* have only been randomly collected by non-closing trawls in the North Atlantic, with all five specimens described appearing morphologically similar [13, 33, 34], which was not in accordance with the multiple types of *L*. *holti* larvae. Until recently however, leptocephali and adult eels have been preserved in formalin, which has prevented attempts to use DNA identification of unknown larvae such as *L*. *holti*. DNA identification is a relatively easy method to match leptocephalus types with their adult species when tissue samples of both forms are available [35–37].

Mitochondrial DNA sequences have proven useful to examine phylogenetic relationships among species, and gene order rearrangements within the mt genome have been demonstrated to be another excellent molecular marker for delimiting phylogenetic relationships of clades [38–40]. The vast majority of vertebrates show a particular mitochondrial gene order (the “canonical” gene order), although when rearrangement events occur within the evolutionary history of a clade, this may result in rearranged gene orders and provide a unique opportunity to resolve genealogies using gene order changes within that given clade. The chance of convergent gene order evolution in highly rearranged mitochondrial genomes is virtually non-existent, especially when multiple rearrangements can be observed [41, 42]. Large-scale gene order rearrangements are present in saccopharyngiforms [3], with different orders observed in closely related families such as Cyematidae and Monognathidae [8]. In fact, the gene order rearrangements in saccopharyngiforms, and especially *Monognathus*, are the most extensive and complicated yet determined in any vertebrate group of taxa and therefore provide exceptional opportunities for reconstructing phylogenetic relationships and/or examining gene duplications, subsequent deletions, and pathways of retaining functional genes.

The present study uses mtDNA sequences to match the *L*. *holti* type of leptocephali from both the Sargasso Sea and the western North Pacific with their adult family (Monognathidae), matches an unusual unknown type of saccopharyngiform leptocephalus collected in the Sargasso Sea in 2011 with a southeast Greenland specimen of *Neocyema* described by Poulsen [13], and examines the phylogeny and gene order rearrangements of these deep-sea eels in relation to other anguilliforms. As a result, we erect a new anguilliform family, the Neocyematidae, whose adults and larvae are now known. We also briefly discuss these new findings in the context of the deep-sea environments where these species live. This now provides a new basis for further research on these unusual deep-sea eels that are rarely collected and are poorly understood ecologically.

## Materials and methods

### Specimens and morphology

A total of 17 specimens of saccopharyngiform fishes (12 leptocephali, 5 adults) are included in the present study and are listed in Table 1 with collection numbers and associated metadata. The adult specimen of *Neoycema* was obtained from off southeast Greenland in 2013 and was described by Poulsen [13] whereas the adults of the other four saccopharyngiform families were included for molecular genetic examinations from the studies of Inoue et al. [3, 8].

**Table 1:** “Saccopharyngiform” materials included in the present study corresponding to materials presented in Fig 5 that all are verified with DNA sequences.

Original ID with photos (Letters in Fig 5)	Tentative species	Specimen	Life stage	Mt genome (prefix AP) or MiFish (prefix LC)	Catch position (degrees)	Region and sampling year	Study
**A.** *Neocyema erythrosoma*	*Neocyema erythrosoma*	ZMUB 21865	Adult	AP018345	64° 25.48N 34° 06.00W	Southeast Greenland 2013	This study
**B.** Unknown Leptocephalus	*Neocyema erythrosoma*	WH342_1248	Larvae	LC315182	25° 04.912N 57° 59.842W	Sargasso Sea 2011	This study
**C**. *Saccopharynx lavenbergi*	*Saccopharynx lavenbergi*	UW 045633	Adult	AB047825	**—**	Eastern Pacific	Inoue et al. [3]
**D.** *Saccopharynx lavenbergi*	*Saccopharynx lavenbergi*	WH342_1580	Larvae	LC315183	27° 00.240N 63° 59.910W	Sargasso Sea 2011	This study
**E.** *Eurypharynx pelecanoides*	*Eurypharynx pelecanoides*	CBM-ZF 10311	Adult	AB046473	**—**	Southern Japan	Inoue et al. [3]
**F.** *Eurypharynx pelecanoides*	*Eurypharynx pelecanoides*	WH404_906	Larvae	LC315184	25° 59.770N 58° 00.080E	Sargasso Sea 2017	This study
**G**. *Cyema atrum*	*Cyema atrum*	**—**	Adult	AP010870	**—**	**—**	Inoue et al. [8]
**H**. *Cyema atrum*	*Cyema atrum*.	WH404_82	Larvae	LC315185	26°29.735N 57°59.849W	Sargasso Sea 2017	This study
**I.** *Monognathus jesperseni*	*Monognathus jesperseni*	**—**	Adult	AP010869	**—**	**—**	Inoue et al. [8]
**J.** *Leptocephalus holti*	*Monognathus jesperseni*	KH-11-6_184	Larvae	LC315186	14° 29.95N 142° 07.65E	NW Pacific 2011	This study
**K**. *Leptocephalus holti*	*Monognathus* sp. #1	MSM41_1404	Larvae	LC315188	26° 00.329N 61° 00.381W	Sargasso Sea 2015	This study
**L.** *Leptocephalus holti*	*Monognathus* sp. #2	WH342_418	Larvae	LC315189	26° 29.852N 63° 59.977W	Sargasso Sea 2011	This study
**No photos available**							
*Leptocephalus holti*	*Monognathus jesperseni*	KH-11-4_52	Larvae	LC315187	12° 40.00N 141° 50.10E	WN Pacific 2011	This study
*Leptocephalus holti*	*Monognathus* sp. #3	WH373_777	Larvae	LC315190	26° 33.24N 69° 59.02W	Sargasso Sea 2014	This study
*Leptocephalus holti*	*Monognathus* sp. #4	WH373_226	Larvae	LC315192	28° 03.78N 69° 57.88W	Sargasso Sea 2014	This study
*Leptocephalus holti*	*Monognathus* sp. #4	WH373_1971	Larvae	LC315191	28° 26.326N 60° 59.467W	Sargasso Sea 2014	This study
*Leptocephalus holti*	*Monognathus* sp. #5	KH-06-1_784	Larvae	LC315193	32° 22.332N 158° 48.165E	Kuroshio extension 2006	This study

The leptocephali consist of eight specimens of *Leptocephalus holti* larvae, one *Cyema atrum*, one *Eurypharynx pelecanoides*, one *Saccopharynx*, and one unknown saccopharyngid larva. The leptocephalus specimens from the Sargasso Sea in the western North Atlantic were collected by an 6.2 m^2^ mouth opening Isaacs-Kidd Midwater Trawl (IKMT) with 0.5 mm mesh during recent efforts to study the abundance and ecology of European eel, *Anguilla anguilla*, leptocephali during their spawning season in March and April of 2011 (WH342 cruise), 2014 (WH373), and 2015 (MSM41) [30, 43]. The western North Pacific specimens were collected by an 8.7m^2^ mouth-opening IKMT with 0.5 mm mesh during efforts to study the spawning area of the Japanese eel, *Anguilla japonica*, in 2011 (KH-11-4 cruise) [44], or by a 5.3 m^2^ mouth-opening MOHT trawl with ~1.5 mm mesh during an interdisciplinary study of the Kuroshio Extension in January-March 2006 (KH-06-1).

Because the leptocephali were collected and identified onboard along with many other species during surveys targeting anguillid larvae, only basic morphological features needed for identification were recorded (size, basic pigmentation, numbers of myomeres etc.) before they were preserved in 99% ethanol or frozen, both of which extensively reduce the availability of detailed morphological information. The exception is the specimen of the unknown saccopharyngid larvae that had a DNA sample taken from its right eye, with the body being preserved in formalin. Therefore, the detailed morphology of most leptocephalus specimens will not be a focus of the present study. The detailed morphologies of the *L*. *holti* type larvae has been examined previously by Smith & Miller [24], as have the other types of larvae [18–19], so therefore is not covered again here. Although there are no voucher specimens available for the Pacific specimens mostly preserved in ethanol, which therefore have lost their body forms, the Sargasso Sea specimens are deposited in the collection of Thuenen Institute of Fisheries Ecology, Hamburg, Germany. No ethical approval was necessary for the present study as DNA tissue samples used were obtained from specimens preserved in museum collections and/or taken onboard previous research cruises.

Nomenclatural Acts: The electronic edition of this article conforms to the requirements of the amended International Code of Zoological Nomenclature, and hence the new name contained herein (Neocyematidae) is available under that Code from the electronic edition of this article. This published work and the nomenclatural acts it contains have been registered in ZooBank, the online registration system for the ICZN. The ZooBank LSIDs (Life Science Identifiers) can be resolved and the associated information viewed through any standard web browser by appending the LSID to the prefix “http://zoobank.org/”. The LSID for this publication is: urn:lsid:zoobank.org:pub:9B2D1B87-A25C-4CC5-8D5C-9127E1A94392. The electronic edition of this work was published in a journal with an ISSN, and has been archived and is available from the following digital repositories: PubMed Central and LOCKSS.

### Molecular genetic analyses

Whole mitogenome DNA sequences were determined for *Neocyema erythrosoma* (ZMUB 21865) and for several additional adult species of notacanthiforms and anguilliforms (Table 1; S1 File). In addition, a fragment of the mitochondrial 12S rRNA gene (MiFish DNA sequence) was determined for all saccopharyngiform adult and larval material available (Table 1). Genomic DNA was extracted using the Qiagen Puregene extraction kit following manufacturer´s protocol and used directly for long and accurate amplification PCR (LA PCR) of the entire mitochondrial genome [45]. Universal fish primers and thermal cycler protocols for the LA PCR were employed according to Miya & Nishida [46]. For the 12S rRNA DNA sequences, MiFish primers and PCR protocols were employed according to Miya et al. [47], in order to amplify a region of approximately 170 base pairs that has been shown to be highly informative for species delimitations when comparing a large number of taxa. The double stranded PCR products were cleaned with Exo-Sap at 60°C for 60 minutes and used as template for direct cycle-sequencing with dye labeled terminators (Applied Biosystems) before sequencing on an automated DNA sequencer. For the taxa determined for their entire mitogenome, the LA PCR fragments were pooled for each species and the mitogenomes were sequenced using next generation sequencing of LA PCR products with the MiSeq Sequencing platform (Illumina) at the Natural History Museum and Institute, Chiba, with all libraries prepared using Nextera XT DNA Library Preparation Kits following the manufacture´s protocol. Briefly, the long PCR products (0.2 ng/μl per sample) were provided for tagmentation, which fragments DNA and then tags the DNA with adapter sequences in a single step. Index 1 (i7), Index 2 (i5), and full adapter sequences were added to the tagmented DNA using a limited-cycle PCR (12 cycles). The resultant library DNA was cleaned up and normalized using AMPure XP beads before MiSeq sequencing (Illumina). Gene annotation was performed using tRNA-scan-SE ver. 1.21 [48] and by alignment to closely related species previously determined for their mitogenomes. Trimming of the MiSeq reads was performed with the MIRA ver. 4 assembler (http://sourceforge.net/p/mira-assembler/wiki/Home/) and assembly of mitogenomes was performed using MITObim with default settings [49] and Sequencher ver. 5.0.1 (Gene codes). Mt gene orders were examined for all five representatives of the saccopharyngiform families. All newly determined mitogenomic DNA sequences were deposited as AP018342–46 in the DDBJ/EMBL/GenBank databases (S1 File). The 13 protein coding gene sequences contained in the mitogenome were aligned by eye for a total of 79 taxa (S2 File) and the MiFish DNA sequences were aligned using ProAlign [50] including only sites with posterior probabilities of 90% or higher (S3 File).

The resultant dataset, including a total of 11,400 base pairs (S2 File), was sectioned into three partitions (-q option) corresponding to the 1^st^, 2^nd^ and 3^rd^ codon positions of the protein coding genes assuming that codon positions experience more similar selection across genes. The two rRNA genes and the 22 tRNA gene sequences present in the mitochondrial genome were excluded due to difficulties with the alignment for saccopharyngiform taxa. The dataset was analyzed using maximum likelihood (ML) method, using the sequential version of the software RAxML ver. 8.1.17 [51]. A single run searching for the best scoring ML-tree, including 1000 bootstrap replicates, was specified using the -f a and–# options, respectively. The model of sequence evolution was the GTR+G+I as found by ModelGenerator ver. 0.85 [52]. Bayesian analysis was performed on the dataset with MrBayes ver. 3.2.6 [53, 54] using the same partitions and models as described above. Convergence of chains and burn-in were determined using Tracer Ver. 1.5 [55].

The 12S rRNA MiFish DNA sequences, including a total of 161 base pairs [47], for the five adult specimens and the 12 saccopharyngiform larvae, including six identified initially as “*Leptocephalus holti”* and one as an unidentified saccopharyngid larva, were obtained to determine the species identity of the unknown larvae and compare the relative similarities and differences among all the different larvae and adults. The sequences were deposited in the above-mentioned repositories with accession numbers LC315182–193 (Table 1). The MiFish DNA sequences were analyzed using uncorrected distances, to construct a NeighborNet network, implemented in SplitsTree4 [56].

## Results and discussion

### *Leptocephalus holti* identified as Monognathidae

**Fig 3:**
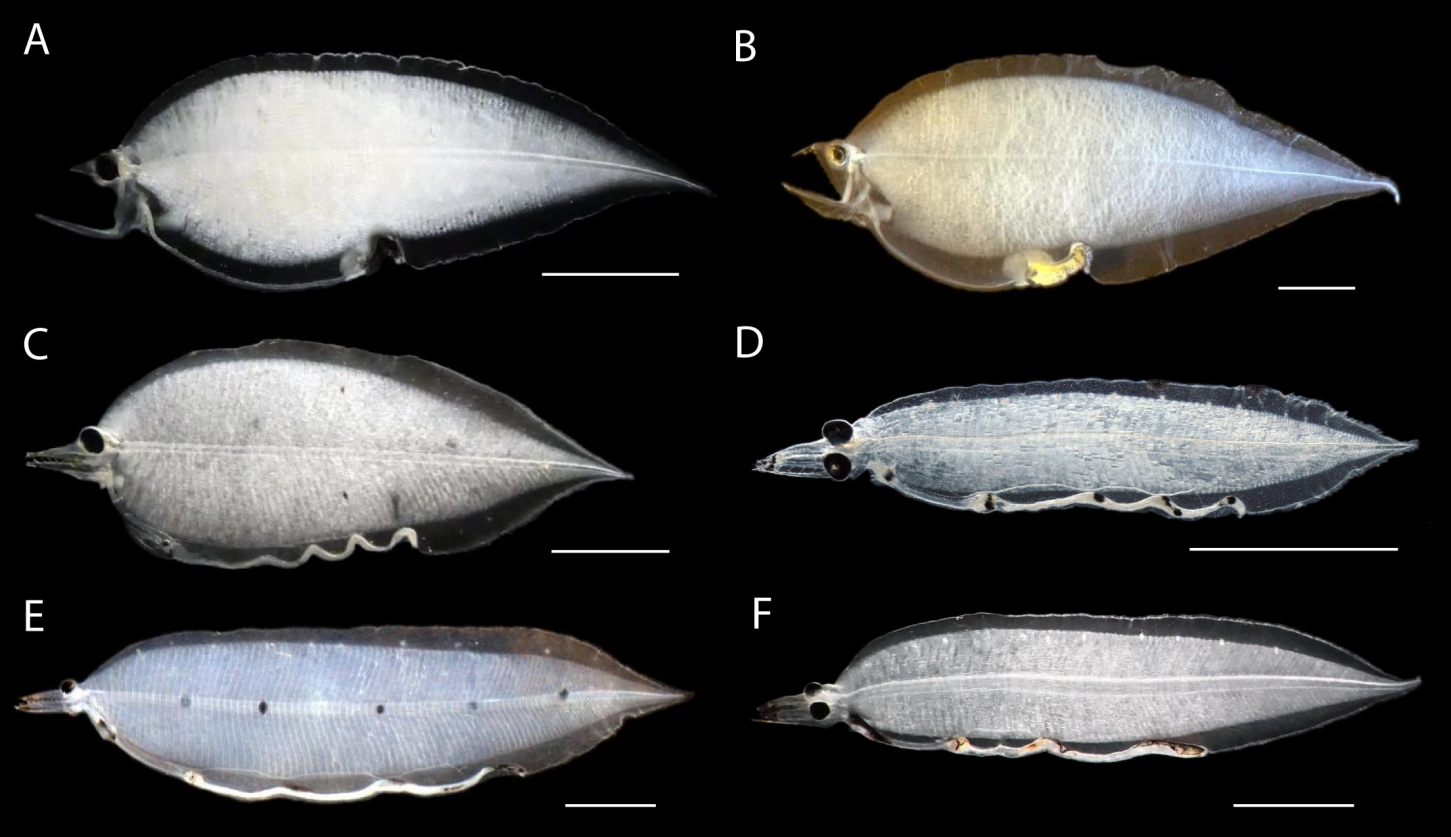
**Leptocephalus larvae of saccopharyngiform fishes known until the present study (A–F).** A, *Eurypharynx* (WH404_906) 25.3 mm. B, *Saccopharynx* (WH342_1580) 40.0 mm. C, *Cyema* (WH404_82) 26.2 mm. D, “*Leptocephalus holti*” Type I (KH-11-6_184) 15.4 mm. E, “*Leptocephalus holti*” Type II (WH342_418) 38.0 mm. F, “*Leptocephalus holti*” Type III (MSM41_1404) 27.1 mm. Scale bars 5 mm.

**Fig 4:**
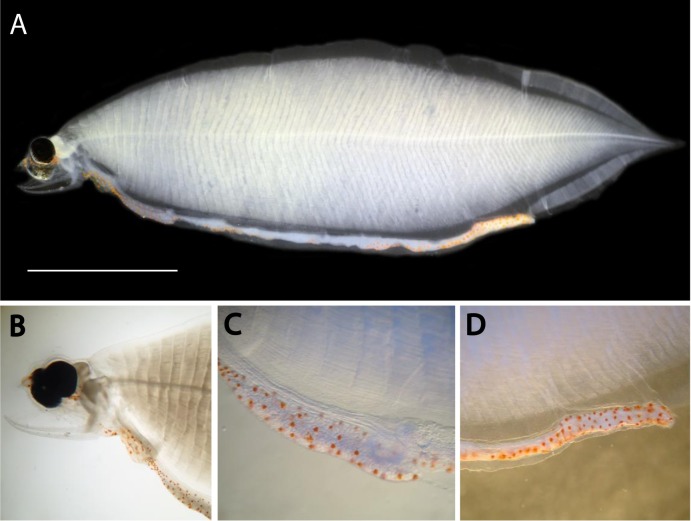
**A Leptocephalus larva (WH342_1248, 22.5 mm) of *Neocyema erythrosoma* from the Sargasso Sea that has unique orange pigment spots (A–D).** A, Whole specimen, 22.5 mm TL. B, Head region. C, Esophagus, liver, stomach region. D, End of the gut region. Scale bar 5 mm.

**Fig 5:**
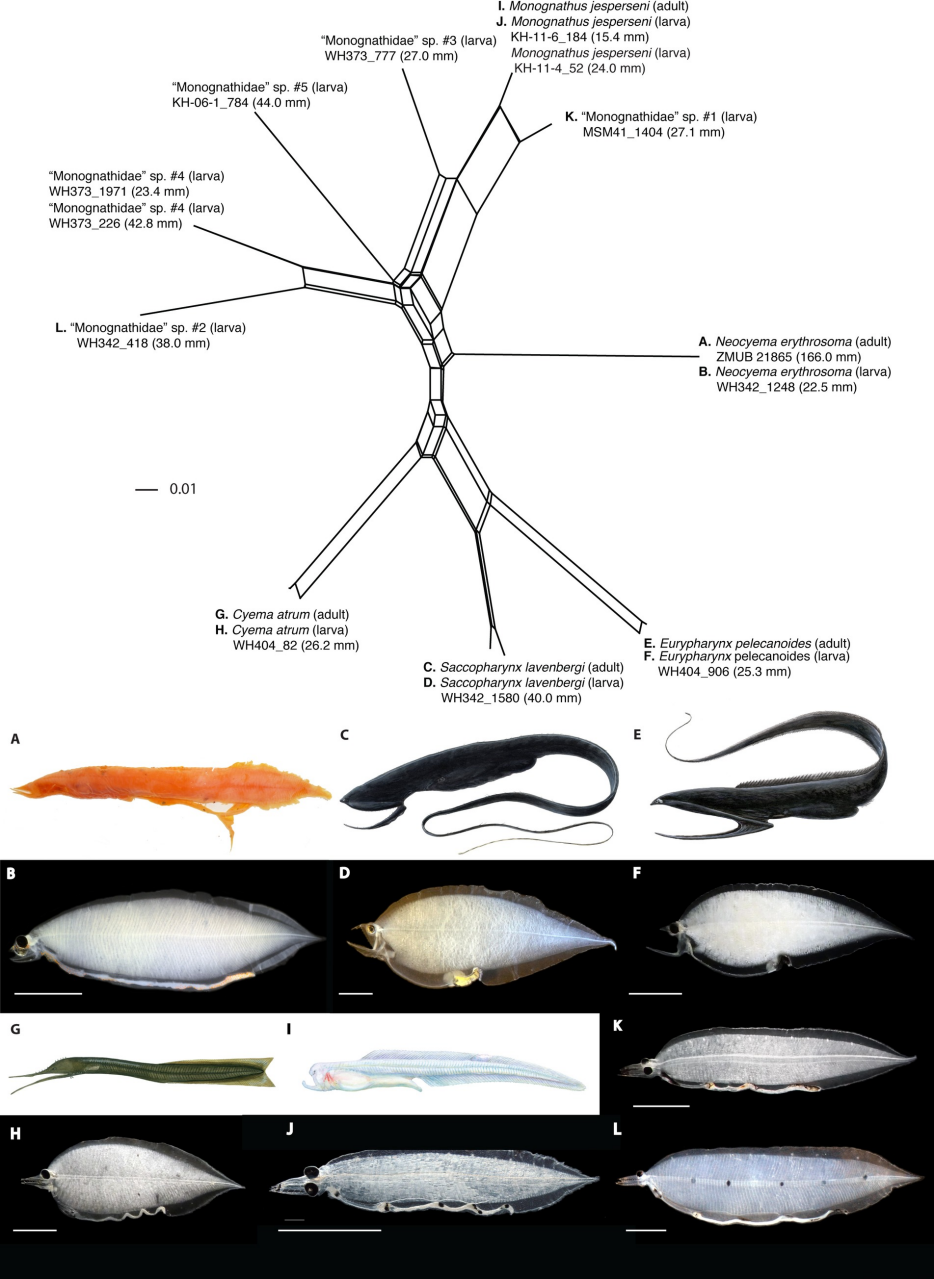
NeighborNet network (uncorrected P-distances) of larvae and adult saccopharyngiforms based on 12S rRNA MiFish DNA sequences. The letters A–M show specimens with DNA sequences of the 12S rRNA MiFish DNA sequences used to construct the network that are matched with photos at the bottom of the figure. General illustrations are shown for adult specimens with no photos (Table 1). Scale bars 5 mm. Although we were able to associate *M*. *jesperseni* with its leptocephalus larvae (I–J), all remaining monognathid leptocephali larvae remain to be associated with adult forms.

The genetic analysis of the MiFish 12S rRNA DNA sequences of eight specimens of *Leptocephalus holti* leptocephali included in this study from both the western North Pacific and Sargasso Sea of the western North Atlantic show for the first time that these are the larvae of the Monognathidae and are not cyematid larvae (Figs 3–5). The MiFish DNA sequences of these larvae were separated into six different species, although photos are available for only three of them (Table 1; Figs 3–5). The multiple species indicated by the MiFish tree is consistent with the at least three major morphological types of *L*. *holti* larvae (referred to here as morphological Types I, II, III) documented by Smith & Miller [24] (referred to as Species 1, 2, 3) and the pigment variation of our specimens. Two of the leptocephali with no lateral pigment and 1–2 dorsal spots (KH-11-4_52, no total myomere (TM) count; KH-11-6_184, 94 TM) (Fig 5) were found to have 100% sequence similarity (MiFish DNA sequences) to an adult specimen of *Monognathus jesperseni* (Fig 5I and 5J) [8]. These appear similar to the *L*. *holti* Type I of Smith & Miller [24]. There was a second species of larvae with just the two dorsal spots (WH373_777, 99 TM) and another species with no lateral pigment and no dorsal spots (MSM41_1404, 100 TM) that is similar to Type III. The Type II larvae that have 4–5 lateral pigment spots and 2–4 dorsal spots includes at least three species according to the MiFish tree, with two being collected in the Sargasso Sea (WH342_418, 114 TM; and WH373_226, ~110 TM; WH373_1971, 93 TM) and one in the western North Pacific (KH-06-1_784, ~127 TM) (Fig 5). The TM of the three types of Smith & Miller [24] heavily overlap with Type I (99–117 TM) being in the lower range, Type III (104–115 TM) being intermediate, and Type II (100–130 TM) being highest. Our TM counts were consistent with those ranges except that the KH-11-6_184 (94 TM) specimen had a lower count than the Type I range, WH373_1971 (93 TM) was lower than the Type II range, and MSM41_1404 (100 TM) was lower than the Type III range. These could reflect actual TM range differences among species within the different types or that our counts are not as accurate as those of the previous studies.

These different types are also consistent with other published collections of the *L*. *holti* type of larvae. The specimen TFMCBMZP 03151 described by De Vera et al. [29] from the Northeastern Atlantic and the two specimens of Fortuño & Olivar [27] from the South Atlantic that both have two dorsal spots and no lateral spots are similar to the Type I and *M*. *jesperseni* from the Pacific and the WH373_777 specimen types from the Sargasso Sea. A leptocephalus specimen noted by Van Utrecht [57] from 37°53´N in the North Atlantic Ocean appears to be a Type II. The Type I and Type II pigmentation types are also reported from the region near Japan [58], and we are not aware of any other specimens that differ from these 3 types being among other specimens we have collected that are not included in the present study.

Only one species of Monognathidae juvenile or adult was available for genetic comparison to our larval specimens (*M*. *jesperseni*), but the morphological variation reported for the later stages likely reflects the variety of species seen among the larvae. Interestingly, Bertelsen & Nielsen [12] speculated about what the larvae of *Monognathus* might be like, and suggested that they would have a “*prolonged suspensorium*, *about 100 myomeres and a series of 4–5 pigment spots on side of body*”. This is consistent with the Type II *L*. *holti* larvae. Raju [26] noted that a specimen (SIO-70-118) showed the lateral pigment spots present on the left side except the last one situated on the right side. Metamorphosing specimens also had 5 lateral pigment spots (Fig 2C) [26]. Bertelsen & Nielsen [12] showed there are two lineages present in *Monognathus*: long-skulled and short-skulled species. There are other distinguishing characters in the described *Monognathus* taxa such as the presence or absence of the pectoral fins and caudal morphology that are as likely as prolonged suspensorium to delimit lineages within this family. The caudal tips of monognathid fishes show three distinct types [12]. Nielsen & Hartel [59] described *Monognathus berteli* showing a caudal filament half the fish SL. The Type I leptocephali were most abundant in the Sargasso Sea in the collections used by Smith & Miller [24], and this type includes the two Pacific larvae identified as *M*. *jesperseni* that appears to be the most common short-skulled one-jaw taxon in the Atlantic Ocean based on available material.

Other comparisons between the larvae and adults can be made as well. For example, the rarity of the *L*. *holti* type of leptocephali may partly be explained by the low fertility of *Monognathus* taxa noted by Bertelsen & Nielsen [12]. It is also clear that the upper jaw is indeed present in the leptocephalus larvae before it is absorbed in the adults, and that the supposedly poisonous fang is not developed in the larvae. Raju [26] showed metamorphic forms of *Monognathus* although in late stages (Fig 2C).

Although there is not enough information presently available to determine which types of *L*. *holti* larvae belong to which types of *Monognathus* adults, a long-standing mystery seems to have been solved. This can be evaluated with further sequencing of the existing specimens and any new specimens that are collected in the future. The inclusions of specimens of Neocyematidae, Eurypharyngidae, Saccopharyngidae, and Cyematidae in the MiFish DNA sequence comparisons (Fig 5) leave little doubt that the *L*. *holti* type of larvae does not belong to any of these taxa, and the perfect match of one of the larvae with *M*. *jesperseni* indicates these are the larvae of the Monognathidae. The number of *L*. *holti* type leptocephali known corresponds with the fact that there are many known types of monognathid adults.

Considering that there is only one genus of that family, it appears that the use of the name “*Leptocephalus holti*” (the genus *Leptocephalus* was applied to unknown species) is no longer justifiable, and that these larvae should be referred to as *Monognathus* spp. after the present study.

### Discovery of *Neocyema* larvae

The MiFish DNA sequence analysis was also successful in resolving the other major mystery among the deep-sea eels, which is the identity of *Neocyema* and their larvae. The similarity of *Neocyema* to *Cyema* and the *L*. *holti* to *Cyema* larvae had created speculation that *L*. *holti* were the larvae of *Neocyema* [18, 24, 33, 34]. However, the MiFish sequence of the *Neocyema* specimen caught off Greenland in the North Atlantic were 100% identical to those of an un-identified saccopharyngid-like leptocephalus from the Sargasso Sea (Figs 4 and 5). The 22.5 mm leptocephalus has jaws that are superficially similar to *Saccopharynx* and has a similarly deep body as that species as well as *Eurypharynx* (Figs 2 and 3) [18]. However, its long straight gut structure is totally different than any of the other leptocephalus types that all have either one large swelling at the end of the gut or multiple gut curvatures (Figs 2–5).

Even more drastically different are the many orange spots on the gut, heart region, and below the eye in the *Neocyema* larvae (Fig 4). A 40 mm leptocephalus (Fig 2D) with a similar body and head shape that was collected in the Sargasso Sea was reported by Castle & Raju [23], who noted it as a yet undescribed genus within the saccopharyngiform fishes, and was included with that group by Smith [18], which also showed many small spots on the posterior region of the gut. That previously collected leptocephalus may have been sorted out of a formalin-preserved plankton sample, and so the orange color of the spots would likely have faded and thus were not reported. Also, the previous specimen was depicted somewhat different from our *Neocyema* leptocephalus (Fig 4), in that it had a straight ventral margin as well as a straight upper jaw (Fig 2D). Those differences could be artifacts of shrinkage during preservation or from damage during collection. Regardless, it is a possibility that the two specimens may be both larvae of *Neocyema* despite the differences observed as our specimen was photographed while fresh before preservation and had some damage to its head. The uniqueness of those spots are consistent with the genetic divergence of the *Neocyema* adult and larvae and that they represent a previously unrecognized family as discussed below.

### Phylogeny and mitochondrial gene orders of the saccopharyngiforms

**Fig 6:**
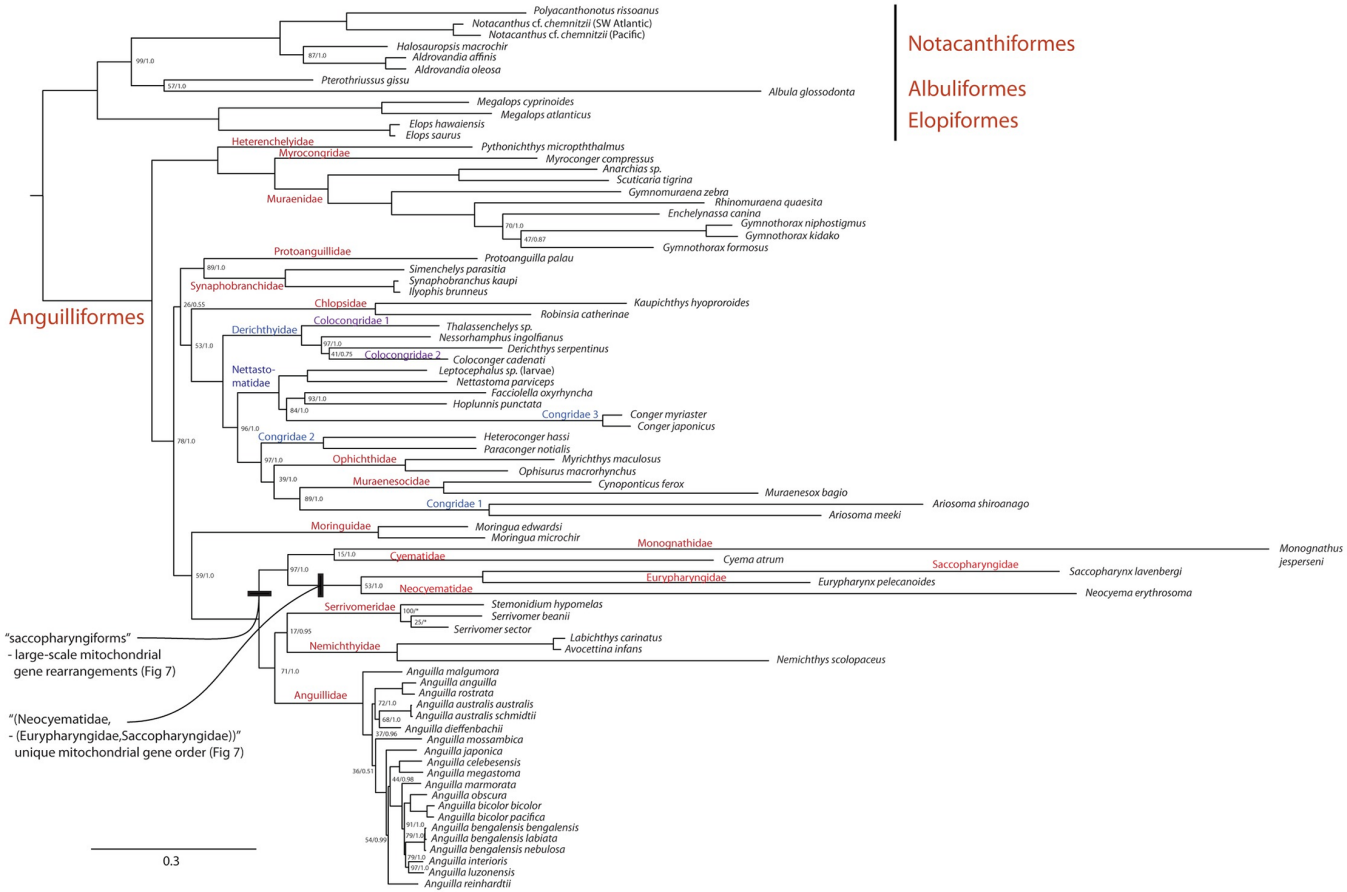
Mitogenomic phylogenetic tree of 79 taxa of the Elopomorpha based on 13 protein-coding genes in the mitochondrial genome (ML and Bayesian analyses, 11,700 base pairs). Bootstrap replicates and Bayesian posterior probability support values for tree nodes are noted only if below 100 and 1.0, respectively. Asterisks (*) denote the only topological difference from the Bayesian analysis compared to the presented ML topology. Notacanthiform, albuliform, and elopiform fishes were used as the outgroups for the Anguilliformes in which saccopharyngiforms constitute a derived clade. Note the extraordinary long branches within saccopharyngiforms, by far the longest within elopomorph fishes. The phylogenetic relationships presented are corroborated by extensive mitochondrial gene orders. Neocyematidae is a sister taxon to the gulper eels (Saccopharyngidae and Eurypharyngidae) and erected as a new family in accordance with gene orders depicted in Fig 7 and morphology, and the families Congridae, Colocongridae and Nettastomatidae are found to be non-monophyletic sensu current classification.

The elopomorph phylogenies (79 taxa), from the ML and Bayesian analyses based on 13 protein coding gene sequences, are presented in Fig 6 including bootstrap and Bayesian posterior probabilities for each node if both values are not 100 or 1.0, respectively. The best scoring ML tree and the majority rule Bayesian consensus tree resulted in identical topologies except for one terminal node within the Serrivomeridae, showing a *Serrivomer sector*–*Stemonidium hypomelas* relationship in the Bayesian analysis (* in Fig 6). The five “saccopharyngiform” families and Serrivomeridae, Nemichthyidae and Anguillidae are shown to comprise a derived subgroup within the elopomorph evolutionary lineage. Neocyematidae is found in a sister position to Eurypharyngidae and Saccopharyngidae with Cyematidae and Monognathidae constituting a clade. The families Congridae, Colocongridae and Derichthyidae were found to be non-monophyletic. The two different OTUs of *Notacanthus* cf. *chemnitzii* corroborate results by Poulsen et al. [60] showing taxonomic uncertainty in this species complex. We have used the name “saccopharyngiforms” throughout because the order “Saccopharyngiformes” is a derived lineage within the Anguilliformes as currently recognized (Fig 6). Therefore, we remove the order Saccopharyngiformes from elopomorph classification. However, the name “saccopharyngiforms” is retained and should be used when referring to the Cyematidae, Neocyematidae, Eurypharyngidae, Saccopharyngidae and Monognathidae, collectively.

**Fig 7:**
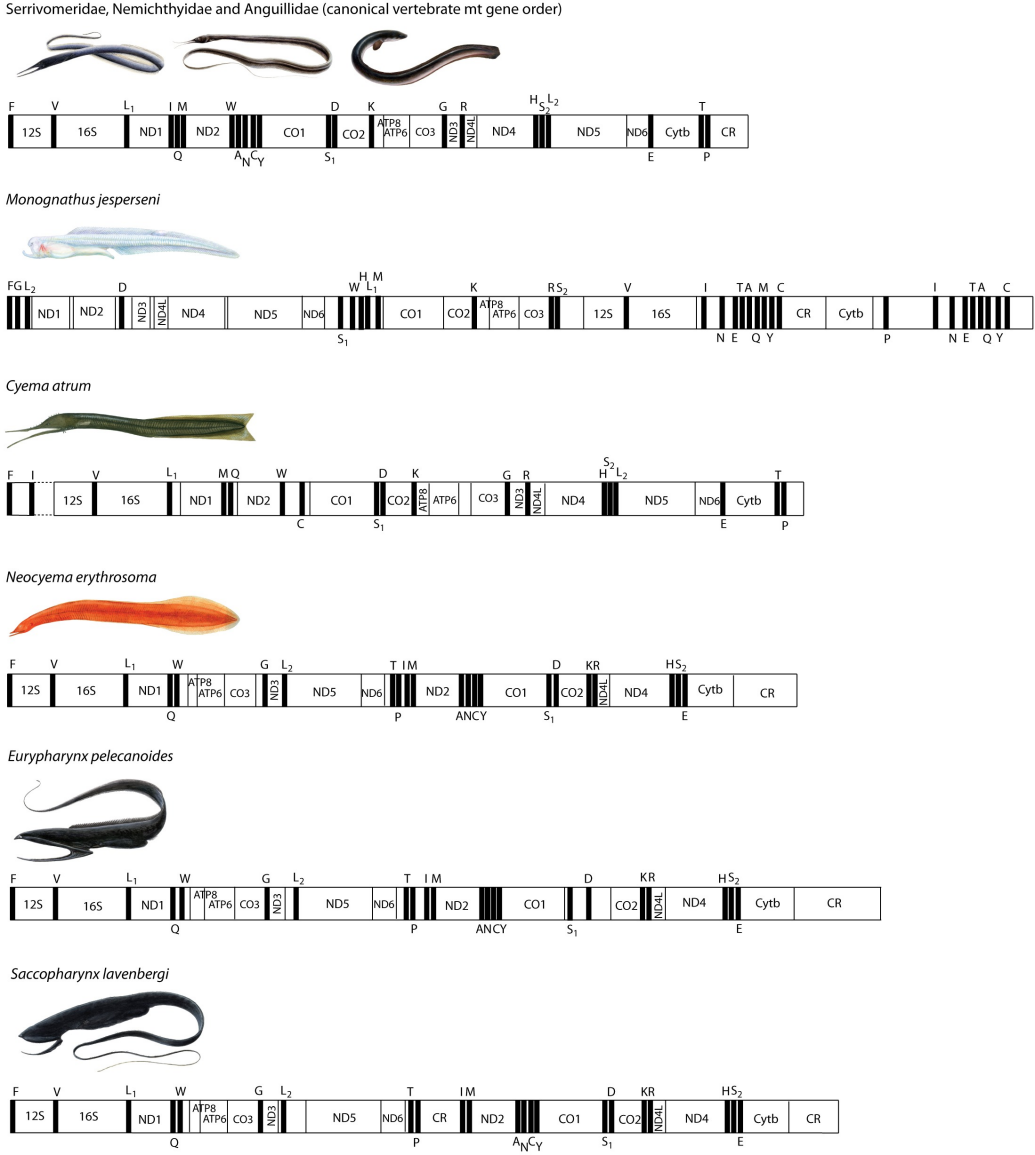
Mitochondrial gene orders of “saccopharyngiform” fishes. Highly similar gene orders are present in *Neocyema*, *Saccopharynx* and *Eurypharynx* compared to *Cyema* and the highly rearranged *Monognathus*, and these orders all differ from the more typical gene orders of the Serrivomeridae, Nemichthyidae, and Anguillidae and other vertebrates.

All saccopharyngiform fishes presently determined for their mt genomes show large-scale gene order rearrangements present (Fig 7) [3, 8]. The newly determined mt genome of *Neocyema* shows a large-scale rearrangement that is highly similar to those observed in *Saccopharynx* and *Eurypharynx* with only small variations. H- and L-strand coding of *C*. *atrum*, *N*. *erythrosoma*, *E*. *pelecanoides* and *S*. *lavenbergi* show the typical H- and L-strand coding (8 tRNAs on the L-strand and 14 tRNAs on the H-strand) whereas *M*. *jesperseni* shows several tRNAs on the H-strand that are usually coded on the L-strand (tRNAs-Ala, -Cys and -Ile). Several tRNA genes show duplicates present and H- and L-strand coding are presented in Fig 7 for all saccopharyngiform taxa included. The mitochondrial gene order of *C*. *atrum* were determined by Inoue et al. [8], although several tRNA genes are missing owing to unknown technical issues. The missing tRNA genes are necessarily located in a region downstream the 12S rRNA (truncated in Fig 7) although not determined by Inoue et al. [8].

The mitochondrial gene order of the newly determined *Neocyema* unambiguously remove it from the Cyematidae family and place it as a sister lineage to the *Saccopharynx*/*Eurypharynx* clade (Fig 7). This is in accordance with the result obtained from the 13 protein-coding gene DNA sequences although the branches involved are exceptionally long and caution should be made in such cases (Fig 6). The similar gene orders observed in *Neocyema*, *Saccopharynx* and *Eurypharynx*, only differing in the presence of various INC-regions throughout the mitochondrial genomes (Fig 7), are strong evidence supporting such a relationship. In fact, considering the very different gene orders observed in *Cyema* and *Monognathus*, and the canonical gene orders present in the Serrivomeridae, Nemichthyidae and Anguillidae (Fig 7), comprising the sister lineage to saccopharyngiforms, similar gene orders as witnessed for the three taxa present an exceptional solid case of monophyly in terms of using gene order rearrangements to clarify evolutionary relationships. Noteworthy, is that the large duplicate Control Region (CR) (992 bp) observed in *S*. *lavenbergi* between tRNAs T-P and I-M is several times smaller in *E*. *pelecanoides* (154 bp) and even shorter in *Neocyema* (37 bp). Inoue et al. [3] noted concerted evolution in the CR fragments in *Saccopharynx*, due to identical CR DNA sequences observed in parts of the fragments. Concerted evolution of CR duplicates is known from several other taxa such as killifishes [61] and snakes [62], and is probably mediated by frequent gene conversions. However, the partial removal of the duplicate CR observed in *Eurypharynx* and *Neocyema* indicates it is a trait that is not selected for in these taxa.

Mitogenomic DNA sequences of saccopharyngiform fishes show the longest branches observed within the Elopomorpha clade and indicate it is an evolutionary history missing much information at present. Our mitogenomic results are largely in accordance with previous studies using mitogenomic data [8, 63] although it is different than a multi-locus DNA study that showed *Cyema* as a sister lineage to the Serrivomeridae and therefore rendered the saccopharyngiform lineage non-monophyletic [64]. The latter result is interesting in terms of *Cyema* showing jaws superficially similar to Nemichthyidae that was resolved as the sister lineage to the Cyematidae-Serrivomeridae relationship. However, this result is not corroborated by mitogenomic DNA sequences, including the gene orders, and not from leptocephalus morphology (Figs 3–5 and 7) [5]. Clearly, longer DNA sequences are important for resolving saccopharyngiform relationships, considering the extraordinary long branch-lengths (Fig 6) [65]. We recognize that long-branch effects in saccopharyngiform fishes potentially could mislead the phylogenetic results (Fig 6) [66–68]. However, the establishment of a new fish family Neocyematidae is based on a similar mitochondrial gene order to the Saccopharyngidae and Eurypharyngidae and the highly different morphologies of the larvae and adult morphotypes (Figs 1, 3 and 4). Short DNA fragments, such as the 12S rRNA DNA sequence, shows an erroneous result with *Eurypharynx* resolved as a sister taxon to *Muraenichthys* [69]. Therefore, Fig 5 demonstrates multiple OTUs (species), and not phylogenetic relationships in saccopharyngiform fishes. The Fig 5 NeighborNet network does, however, show large variation in monognathids and saccopharyngiforms as also demonstrated by the leptocephali and mt gene orders.

### The new family of Neocyematidae

All morphological and genetic evidence presented here support the establishment of a new family, Neocyematidae fam. nov., that has a phylogenetic affinity with the gulper- and swallower eels as demonstrated by their deep-bodied larvae and 11,400 base pairs of mitogenomic DNA sequences and gene orders (Figs 1–7). The unusual and unique larva collected in the Sargasso Sea (Fig 4) was 100% identical in its MiFish DNA sequences to the *Neocyema* adult collected off southeast Greenland (Fig 5A and 5B). *Neocyema* was clearly a distinct taxon in the phylogenetic tree, with a distinct gene order, and with a distinct leptocephalus larva. The genetic divergence between *Neocyema* and other saccopharyngiform families is greater than among other anguilliform families, which in combination with the morphological differences seem to leave little doubt about its distinct family-level status, despite only one species being known so far. The establishment of a new family Neocyematidae is especially supported when comparing morphological differences between *Neocyema* and the two families of which it shares large-scale rearranged gene orders; Saccopharyngidae and Eurypharyngidae. These two families show more similar morphologies compared to *Neocyema* and family status for the monotypic *Neocyema* is therefore appropriate. Interestingly, this is the second new anguilliform family to be erected recently, after *Protoanguilla palau* (Fig 6) was discovered living in underwater caves in Palau, which resulted in the establishment of the family Protoanguillidae [63]. However, the larvae of *P*. *palau* remain to be discovered.

*Neocyema* is now known from five described adults and from a 22.5 mm larva, but no post-leptocephalus metamorphosing specimens have been collected yet. Considering the rarity of these fish in collections, metamorphosing specimens may not be collected in the near future. However, several peculiar features are present that must change during the transformation from larva to adult that are different than the characteristics of other saccopharyngiforms. The eye (large in the larvae) becomes reduced/grown over in the adult (likely blind), which is a greater reduction compared to other species relative to in the larvae. More striking is that the larvae have orange pigmentation on the gut and head regions, with the adult being completely red/orange, and the larvae show no black pigmentation, a common feature in other saccopharyngiforms. In addition, the larvae show head morphology somewhat reminiscent of *Eurypharynx* and *Saccopharynx* although the adult transforms into a superficial body shape more similar to *Cyema*. The removal of *Neocyema* from the family Cyematidae is un-surprising, as the only character that supported a *Cyema*-*Neocyema* relationship was the superficial short body form, which was also noted as problematic by Poulsen [13]. Similarly, the semi-extended jaws in *Neocyema* are unique among saccopharyngiforms clearly distinguishing it from *Cyema* that shows jaws superficially reminiscent of that observed in nemichthyids (Fig 7).

### Morphology and ecology of “saccopharyngiform” fishes

Saccopharyngiform fishes are strange deep-sea fishes that are predators of the lower mesopelagic and bathypelagic zones where they are only rarely collected in trawls that fish at such great depths. There is no light at the great depths of the bathypelagic zone (>1000 m) except for that generated through bioluminescence and chances of capturing prey are likely widely spaced in time [70]. Interestingly, the 28 *Monognathus rosenblatti* eels collected from 600–1600 m off the bottom at depths of 4200–5200 m at 31°N, 159°W in the central North Pacific made it the most abundant species of pelagic fish or invertebrate collected by trawl [71]. Two *Monognathus smithi* were also collected there at 4200 m, but no other anguilliform fishes were found at those great depths. *Monognathus jesperseni* has been collected in mid-water at a shallower depth ≤1592 m, as has *Neocyema* at ≤2284 m [72] or at 1600–2300 m [33, 34], and a few specimens of some *Monognathus* species have been collected between about 100–1000 m in addition to the deeper depths [12]. The *Neocyema* eel specimen from off Greenland included in this study was likely caught in pelagic waters by getting entangled in the mesh of the trawl (found on the deck, not in the cod-end) before or after bottom-trawling occurred at depths of about 1180 m [13]. *Cyema atrum*, *Eurypharynx pelecanoides* and *Saccopharynx* eels have also been collected at depths shallower than 3000 m [9, 73], with *E*. *pelecanoides* being collected between 600 and 2300 m in the Atlantic [11]. Whether saccopharyngiforms may use diel vertical migration (DMV) behavior to move to shallower depths at night for feeding, and back to deeper depths during the day to avoid predation, is not known, although some anguilliform leptocephali appear to use DMV [74]. This might be advantageous for saccopharyngiforms in the shallower depth ranges where some species live, but the morphology of these species do not suggest they are particularly strong swimmers. The number of pelagic fish species drop off steadily with depth [75], so the unique morphological features of saccopharyngiform fishes are likely related to selective pressures associated with adapting to the unique environment of the deep-sea.

These fishes show exceptional modifications that include extreme fusion and reductions of cranial and pectoral skeletons across all five families compared to other anguilliform fishes supporting the clade as a monophyletic lineage. Monognathid adults have only the skull, suspensorium and lower jaw in the head-region as well as some pectoral elements [76–78], a feature that is also modified across the saccopharyngiform families [13, 33, 79]. A transformation of the vertebral column occurs as noted in works on both the Monognathidae [12] and *Neocyema* [13, 33] because adults were observed to have non-ossified vertebral columns. The morphology of the caudal region is exceptionally different among saccopharyngiform fishes, being simple in *Neocyema*, forked in *Cyema*, and long and thin in *Eurypharynx* and *Saccopharynx* (Figs 1 and 5) [78], and it also varies among species within Monognathidae [12] and for Saccopharyngidae [10]. For example, monognathids show no caudal fin rays although there are differences in their caudal regions, with one species, *Monognathus berteli*, having a long caudal filament [59].

The long caudal region with a luminous organ at the end in *Eurypharynx* and *Saccopharynx* [80] could be used as a lure to attract prey as the luminous lures are thought to be used in some deep-sea fishes such as lophiiform anglerfishes and stomiiform dragonfishes [81]. The apparently poisonous rostral fangs present in the Monognathidae appear to be for immobilizing large shrimp that are grasped in their jaws [12]. It has also been speculated that monognathids have glands that may release odors to attract the shrimp, since the monognathids lack well developed sensory systems to search for prey, and that because of their small size of mostly <85 mm and a maximum size of 159 mm, they might prepare for reproduction after one or a few meals on large shrimp at sizes starting about 50 mm [12]. The gulper eels reach larger sizes up to at least about 1.5 m, and *Eurypharynx* have more diverse generalist diets feeding on fishes, crustaceans and squid using their large expandable jaws designed to eat very large prey relative to their body size [11, 70], although *Saccopharynx* appears adapted to ingest even larger prey than *Eurypharynx* [78]. The diet of *Cyema atrum* is unknown, but they have sensory pores and papillae on their head and body that could be used to detect prey [9]. All of the saccopharyngiforms are one-time spawners [12], with sexual dimorphism occurring in *Eurypharynx* and *Saccopharynx* [11, 78]. These various morphological, behavioral, and reproductive characteristics indicate these deep-sea eels have evolved interesting ecological niches in the deep light-free zones of the seas, but their larvae live in the upper 300 m of the ocean. The existence of 14 species of monognathids (most species described by Bertelsen & Nielsen [12]), some of which may be among the most abundant species in the deepest bathypelagic zones according to one study [71], suggests that the group underwent a rarely considered species radiation where few other pelagic fish species are known to live.

### Distributions of saccopharyngiform fishes

*Neocyema* has presently only been documented from the North and South Atlantic Ocean, whereas Monognathidae, Cyematidae and Saccopharyngidae have been reported from all major oceans [2, 10, 12]. *Eurypharynx pelecanoides* is currently considered monotypic for the Eurypharyngidae and is a relatively commonly collected in the Atlantic and Pacific Oceans. It is frequently collected as leptocephali in the Sargasso Sea where it spawns based on the presence of small larvae [82]. However, *Cox1* barcoding results examined in the present study shows two clearly delimited OTUs present among specimens identified as *E*. *pelecanoides* that have no associations with ocean distributions (S4 File). *Eurypharynx* has been used in species distribution modeling due to its low chance of misidentification [83], although two OTUs as observed in this study illustrates the need for more taxonomic research on both adult and larval “saccopharyngiform” fishes. *Neocyema* leptocephali and adults are extraordinary rare and so far, have only been collected in the Atlantic Ocean. Five adult specimens have been reported before to this study [13, 33, 34], although a sixth (NMS.Z.2010.85.1) has also been caught in 2009 in the North Atlantic Ocean at approximately 49°46N, 27°50W with a fishing depth of approximately 2750 m during the ECOMAR cruise JC037. That specimen was preserved in formalin with no tissue sample available (Fig 1A). Both the *Neocyema* leptocephalus presented in this study (Fig 4) and the similar specimen described by Castle & Raju [23] and reproduced by Smith [18] shown in Fig 2D were collected in the Sargasso Sea.

### Conclusions

The deep-sea pelagic eels of the monophyletic lineage of saccopharyngiform fishes are shown to have evolved to consist of at least five distinct living families that are corroborated by their leptocephalus and adult morphotypes in combination with mitogenomic DNA sequence data for all families. Mitochondrial gene orders are exceptionally informative in the case of saccopharyngiform fishes as all five families show unique gene orders. A new family Neocyematidae is erected based partly on a newly discovered leptocephalus specimen from the Sargasso Sea that has an identical DNA sequence as an adult specimen from off Greenland, which is one of only six adult specimens presently known. Mitogenomic DNA sequences and extensive gene order rearrangements similarly support the establishment of a new family. We show that the “*Leptocephalus holti*” larval types previously considered as possibly being the larvae of *Neocyema* are in fact, the larvae of the one-jaw family Monognathidae. These leptocephali from the Atlantic and Pacific show variability in pigmentation patterns and constitute at least 6 different species that are not yet possible to associate with adult specimens, with the notable exception of *Monognathus jesperseni*. These findings make it possible to attribute future collected larval and adult specimens to the correct saccopharyngiform families and trace unique gene orders within this elusive saccopharyngiform deep-sea lineage.

## Supporting information

S1 File List of elopomorph mitochondrial genomes (79 taxa) used in the present study. (DOCX)

S2 File By-gene alignment of 13 protein coding genes in the mitochondrial genome of 79 elopomorph taxa analyzed in the present study. Phylogenetic tree presented in Fig 6.
(NEX)

S3 File 12S rRNA (MiFish DNA sequences) alignment of 17 saccopharyngiform adults and larvae. Phylogenetic network presented in Fig 5.
(NEX)

S4 File *Cox1* (cytochrome oxidase subunit 1) neighbor-joining phylogenetic tree of *Eurypharynx* generated from the Barcoding of Life Database (BOLD, www.barcodinglife.org). Materials and methods are presented in Poulsen et al. [60] and Greenland records (GLF records) can be found in the BOLD repository under the Greenland Fishes Barcoding Project (Poulsen et al.) [60]. Note how the two OTUs of *Eurypharynx* cf. *pelecanoides* show no associations with geography of samples.
(TIF)

## References

[1] Böhlke EB, editor. Leptocephali Fishes of Western North Atlantic. Sears Foundation for Marine Research, New Haven 1989a; 9(2). pp. 657–1055.

[2] Smith DG. Introduction to leptocephali In: Böhlke EB, editor. Fishes of the Western North Atlantic. Sears Foundation for Marine Research, New Haven, Memoir 1 1989a; 1(*9*). pp. 657–668.

[3] Inoue JG, Miya M, Tsukamoto K, Nishida M. Evolution of the deep-sea gulper eel mitochondrial genomes: large-scale gene rearrangements originated within the eels. Mol Biol Evol. 2003; 20(11): 1917–24. 10.1093/molbev/msg206 12949142

[4] Inoue JG, Miya M, Tsukamoto K, Nishida M. Mitogenomic evidence for the monophyly of elopomorph fishes (Teleostei) and the evolutionary origin of the leptocephalus larva. Mol Phylogenet Evol. 2004; 32: 274–286. 10.1016/j.ympev.2003.11.009 15186813

[5] Miller MJ, Tsukamoto K. An introduction to leptocephali: biology and identification 1st ed Tokyo: Ocean Research Institute, University of Tokyo; 2004; pp. 1–96.

[6] Miller MJ. Ecology of anguilliform leptocephali: remarkable transparent fish larvae of the ocean surface layer. Aqua-BioSci Monogr (SBSM). 2009; 2(4): 1–94.

[7] Böhlke EB, editor. Fishes of the Western North Atlantic Sears Foundation for Marine Research, New Haven 1989b; 9(2). pp. 1–655.

[8] Inoue JG, Miya M, Miller MJ, Sado T, Hanel R, Hatooka K, Aoyama J, Minegishi Y, Nishida M, Tsukamoto K. Deep-ocean origin of the freshwater eels. Biol Lett. 2010; 6: 363–366. 10.1098/rsbl.2009.0989 20053660 PMC2880065

[9] Smith DG. Family Cyematidae In: Böhlke EB, editor. Fishes of the Western North Atlantic. Sears Foundation for Marine Research, New Haven, Memoir 1 1989b; 9(1). pp. 630–635.

[10] Nielsen JG, Bertelsen E. The gulper-eel family Saccopharyngidae (Pisces, Anguilliformes). Steenstrupia. 1985; 11(6): 157–206.

[11] Nielsen JG, Bertelsen E, Jespersen Å. The biology of *Eurypharynx pelecanoides* (Pisces, Eurypharyngidae). Acta Zoologica. 1989; 70(3): 187–197.

[12] Bertelsen E, Nielsen JG. The deep sea eel family Monognathidae (Pisces, Anguilliformes). Steenstrupia. 1987; 13: 141–198.

[13] Poulsen JY. Fifth confirmed record and North Atlantic range expansion of the rare pelagic bobtail snipe eel genus *Neocyema* (Cyematidae, Elopomorpha). Mar Biodiv Rec. 2015; 8(e53): 1–5. 10.1017/S175526721500024X

[14] Nelson JS. Fishes of the world, 4th ed Hoboken, NJ: John Wiley & Sons; 2006.

[15] Robins CR. The phylogenetic relationships of the anguilliform fishes. Orders Anguilliformes and Saccopharyngiformes In: Böhlke EB, editor. Fishes of the Western North Atlantic. Sears Foundation for Marine Research New Haven, CT: 1989; 1. pp. 9–23.

[16] Johnson GD, Paxton JR, Sutton TT, Satoh TP, Sado T, Nishida M, Miya M. Deep-sea mystery solved: astonishing larval transformations and extreme sexual dimorphism unite three fish families. Biol Lett. 2009; 5: 235–239. 10.1098/rsbl.2008.0722 19158027 PMC2667197

[17] Poulsen JY, Sado T, Hahn C, Byrkjedal I, Moku X, Miya M. Preservation obscures pelagic deep-sea fish diversity: Doubling the number of sole-bearing opisthoproctids and resurrection of the genus *Monacoa* (Opisthoproctidae, Argentiniformes). PloS ONE. 2016; 11(8): e0159762 10.1371/journal.pone.0159762 27508419 PMC4980007

[18] Smith DG. Family Cyematidae: Leptocephali In: Böhlke EB, editor. Fishes of the Western North Atlantic. Sears Foundation for Marine Research, New Haven, Memoir 1; 1989c; 9(2). pp. 944–947.

[19] Smith DG. Saccopharyngidae, Eurypharyngidae, and Monognathidae: Leptocephali In: Böhlke EB, editor. Fishes of the Western North Atlantic. Sears Foundation for Marine Research, New Haven, Memoir 1; 1989d 9(2). pp. 948–954.

[20] Smith DG. Unidentified Leptocephali In: Böhlke EB, editor. Fishes of the Western North Atlantic. Sears Foundation for Marine Research, New Haven, Memoir 1; 1989e 9(2). pp. 973–981.

[21] Smith DG.Guide to the leptocephali (Elopiformes, Anguilliformes, and Notacanthiformes) NOAA Tech Rep 1979; NMFS Circ. 424. pp. 1–39. http://spo.nmfs.noaa.gov/Circulars/CIRC424.pdf.

[22] Orton GL. Notes on larval anatomy of fishes of the order Lyomeri. Copeia. 1963; 1: 6–15.

[23] Castle PHJ, Raju NS. Some rare leptocephali from the Atlantic and Indo-Pacific Oceans. Dana Rep. 1975; 85: 1–25.

[24] Smith DG, Miller MJ. Cyematid larvae of the *Leptocephalus holti* group in the Atlantic and Pacific Oceans (Pisces: Saccopharyngiformes). Breviora; 1996; 503: 1–12.

[25] Schmidt J. On the occurrence of leptocephali (larval muraenoids) in the Atlantic W. of Europe. Meddelelser fra Kommissionen for Havundersøgelser, Serie Fiskeri. 1909; 3: 1–19.

[26] Raju SN. Three new species of the genus *Monognathus* and the leptocephali of the order Saccopharyngiformes. Fish Bull. 1974; 72: 547–562.

[27] FortuñoFour different larval morphotypes are currently known in Neocyema and Monognathidae and shown in Figure 1. associaiton ed with JM, Olivar MP. Larvas de Anguilliformes capturadas en el Atlántico sudoriental. Misc Zool. 1986; 10: 223–231.

[28] Miller MJ, Aoyama J, Mochioka N, Otake T, Castle PH, Minagawa G, Inagaki T, Tsukamoto K. Geographic variation in the assemblages of leptocephali in the western South Pacific. Deep Sea Res Part I: Oceanographic Research Papers. 2006; 53(5): 776–794. 10.1016/j.dsr.2006.01.008

[29] De Vera A, Hernández F, Burgos E. Datos sobre la presencia de una larva de Cyematidae en el océano Atlántico oriental (Pisces: Saccopharyngiformes). Vieraea: Folia scientarum biologicarum canariensium. 2014; 42: 179–186.

[30] Miller MJ, Stepputtis D, Bonhommeau S, Castonguay M, Schaber M, Vobach M, Wysujack K, Hanel R. Comparisons of catches of large leptocephali using an IKMT and a large pelagic trawl in the Sargasso Sea. Mar Biodiv. 2013; 43(4): 493–501. 10.1007/s12526-013-0170-7

[31] Miller MJ, Feunteun E, Aoyama J, Watanabe S, Kuroki M, Lecomte-Finiger R, Minegishi Y, Robinet T, Réveillac E, Gagnaire PA, Berrebi P. Biodiversity and distribution of leptocephali west of the Mascarene Plateau in the southwestern Indian Ocean. Prog Oceanogr. 2015; 137: 84–102. 10.1016/j.pocean.2015.05.026

[32] Wouthuyzen S, Miller MJ, Aoyama J, Minagawa G, Sugeha HY, Suharti SR, Inagaki T, Tsukamoto K. Biodiversity of anguilliform leptocephali in the central Indonesian Seas. Bull Mar Sci. 2005; 77(2): 209–24.

[33] Castle PHJ. A new genus and species of bobtail eel (Anguilliformes, Cyemidae) from the South Atlantic. Arch Fischerei. 1977; 28: 69–76.

[34] DeVaney SC, Hartel KE, Themelis DE. The first records of *Neocyema* (Teleostei: Saccopharyngiformes) in the Western North Atlantic with comments on its relationship to *Leptocephalus holti* Schmidt 1909. Northeastern Naturalist. 2009; 16: 409–414.

[35] Ma T, Miller MJ, Aoyama J, Tsukamoto K. Genetic identification of *Conger myriaster* leptocephali in East China Sea. Fish Sci. 2007; 73: 989–94. 10.1111/j.1444-2906.2007.01427.x

[36] Tawa A, Kobayakawa M, Yoshimura T, Mochioka N. Identification of leptocephali representing four muraenid species from the western North Pacific, based on morphometric and mitochondrial DNA sequence analyses. Bull Mar Sci. 2013; 89(2): 461–81. 10.5343/bms.2012.1010

[37] Anibaldi A, Benassi Franciosi C, Massari F, Tinti F, Piccinetti C, Riccioni G. Morphology and species composition of southern Adriatic Sea leptocephali evaluated using DNA barcoding. PLoS ONE. 2016; 11(11): e0166137 10.1371/journal.pone.0166137 27893773 PMC5125788

[38] Desjardins P, Morais R. Sequence and gene organization of the chicken mitochondrial genome: a novel gene order in higher vertebrates. J Mol Biol. 1990; 212: 599–634. 10.1016/0022-2836(90)90225-B 2329578

[39] Satoh TP, Miya M, Endo H, Nishida M. Round and pointed-head grenadier fishes (Actinopterygii: Gadiformes) represent a single sister group: Evidence from the complete mitochondrial genome sequences. Mol Phylogenet Evol. 2006; 40: 129–138. 10.1016/j.ympev.2006.02.014 16603389

[40] Okajima Y, Kumazawa Y. Mitochondrial genomes of acrodont lizards: Timing of gene rearrangements and phylogenetic and biogeographic implications. BMC Evol Biol. 2010; 10: 141 10.1186/1471-2148-10-141 20465814 PMC2889956

[41] Boore JL, Brown WM. Big trees from little genomes: mitochondrial gene order as a phylogenetic tool. Curr Opinion in Gen Dev. 1998; 8: 668–674. 10.1016/s0959-437x(98)80035-x 9914213

[42] Poulsen JY, Byrkjedal I, Willassen E, Rees D, Takeshima H, Satoh TP, Shinohara G, Nishida M, Miya M. Mitogenomic sequences and evidence from unique gene rearrangements corroborate evolutionary relationships of myctophiformes (Neoteleostei). BMC Evol Biol. 2013; 13(111): 1–21. 10.1186/1471-2148-13-111 23731841 PMC3682873

[43] Hanel R, Stepputtis D, Bonhommeau S, Castonguay M, Schaber M, Wysujack K, Vobach M, Miller MJ. Low larval abundance in the Sargasso Sea: new evidence about reduced recruitment of the Atlantic eels. 2014; Naturwissenschaften. 10.1007/s00114-014-1243-6 25307845

[44] Aoyama J, Watanabe S, Miller MJ, Mochioka N, Otake T, Yoshinaga T, Tsukamoto K. Spawning sites of the Japanese eel in relation to oceanographic structure and the West Mariana Ridge. PLoS One. 2014; 9(2): e88759 10.1371/journal.pone.0088759 24551155 PMC3923831

[45] Cheng S, Higuchi R, Stoneking M. Complete mitochondrial genome amplification. Nat Gen. 1994; 7: 350–51. 10.1038/ng0794-350 7920652

[46] Miya M, Nishida M. Organization of the mitochondrial genome of a deep-sea fish, *Gonostoma gracile* (Teleostei: Stomiiformes): first example of transfer RNA gene rearrangements in bony fishes. Mar Biotech. 1999; 1(5): 416–26. 10.1007/PL00011798 10525676

[47] Miya M, Sato Y, Fukunaga T, Sado T, Poulsen JY, Sato K, Minamoto T, Yamamoto S, Yamanaka H, Araki H, Kondoh M. MiFish, a set of universal PCR primers for metabarcoding environmental DNA from fishes: detection of more than 230 subtropical marine species. Royal Soc Open Sci. 2015; 2(7): 150088 10.1098/rsos.150088 PMC4632578 26587265

[48] Schattner P, Brooks AN, Lowe TM. The tRNAscan-SE, snoscan and snoGPS web servers for the detection of tRNAs and snoRNAs. Nuc Acids Res. 2005; 33: W686–689. 10.1093/nar/gki366 PMC1160127 15980563

[49] Hahn C, Bachmann L, Chevreux B. Reconstructing mitochondrial genomes directly from genomic next-generation sequencing reads–a baiting and interative mapping approach. Nuc Acids Res. 2013; 41(e129): 1–9. 10.1093/nar/gkt371 PMC3711436 23661685

[50] Löytynoja A, Milinkovitch MC. A hidden Markov model for progressive multiple alignment. Bioinformatics. 2003; 19(12): 1505–13. 10.1093/bioinformatics/btg193 12912831

[51] Stamatakis A. RAxML Version 8: A tool for Phylogenetic Analysis and Post-Analysis of Large Phylogenies. Bioinformatics. 2014; 1–2. 10.1093/bioinformatics/btu033 PMC3998144 24451623

[52] Keane TM, Creevey CJ, Pentony MM, Naughton TJ, McInerney JO. Assessment of methods for amino acid matrix selection and their use on empirical data shows that ad hoc assumptions for choice of matrix are not justified. BMC Evol Biol. 2006; 6: 29 10.1186/1471-2148-6-29 16563161 PMC1435933

[53] Huelsenbeck JP, Ronquist F. MRBAYES: Bayesian inference of phylogeny. Bioinformatics. 2001; 17: 754–55. 11524383 10.1093/bioinformatics/17.8.754

[54] Ronquist F, Huelsenbeck JP. MRBAYES 3: Bayesian phylogenetic inference under mixed models. Bioinformatics. 2003; 19: 1572–74. 12912839 10.1093/bioinformatics/btg180

[55] Rambaut A, Suchard MA, Xie D, Drummond AJ. Tracer v1.6. 2014. Available: http://tree.bio.ed.ac.uk/software/tracer/.

[56] Huson DH, Bryant D. Application of Phylogenetic Networks in Evolutionary Studies. Mol Biol Evol. 2006; 23(2): 254–67. 10.1093/molbev/msj030 16221896

[57] Van Utrecht WL. A distinctive leptocephalus from the Mid-North Atlantic. Copeia. 1987; 2: 517–9.

[58] Mochioka N, Tabeta O. Leptocephali In: Okiyama M, editor. An Atlas of the Early Stage of Fishes in Japan, 2nd ed Minamiyama: Tokai University Press; 2014 pp. 2–89 (in Japanese).

[59] Nielsen JG, Hartel KE. *Monognathus berteli* sp. nov. from the Indian Ocean (Pisces, Monognathidae). Ichthyol Res. 1996; 43(2): 113–5.

[60] Poulsen JY, Thorkildsen S, Hammeken NA. Identification keys to halosaurs and notacanthids (Notacanthiformes, Elopomorpha) in the subarctic North Atlantic Ocean including three new species records and multiple molecular OTUs of *Notacanthus* cf. *chemnitzii*. Mar Biodiv. 2018; 48(2): 1009–1025. 10.1007/s12526-017-0762-8

[61] Tatarenkov A, Avise JC. Rapid concerted evolution in animal mitochondrial DNA. Proc Royal Soc London B: Biol Sci. 2007; 274: 1795–8. 10.1098/rspb.2007.0169 PMC2493574 17490947

[62] Kumazawa Y, Ota H, Nishida M, Ozawa T. The complete nucleotide sequence of a snake (*Dinodon semicarinatus*) mitochondrial genome with two identical control regions. Genetics. 1998; 150(1): 313–29. 9725849 10.1093/genetics/150.1.313 PMC1460336

[63] Johnson GD, Ida H, Sakaue J, Sado T, Asahida T, Miya M. A `living fossil`eel (Anguilliformes: Protoanguillidae, fam. nov.) from an undersea cave in Palau. Proc R Soc B. 2012; 279(1730). 10.1098/rspb.2011 PMC3259923 21849321

[64] 1289Santini F, Kong X, Sorenson L, Carnevale G, Mehta RS, Alfaro ME. A multi-locus molecular timescale for the origin and diversification of eels (Order: Anguilliformes). Mol phylogenet evol. 2013; 69(3): 884–94. 10.1016/j.ympev.2013.06.016 23831455

[65] Inoue JH, Miya M. Phylogeny of the basal teleosts, with special reference to the Elopomorpha. Japan J Ichthyol. 2001; 48(2): 75–91 (in Japanese).

[66] Felsenstein J. Cases in which parsimony or compatibility methods will be positively misleading. Syst Zool. 1978; 27: 401–10.

[67] Bergsten J. A review of long-branch attraction. Cladistics. 2012; 21(2): 163–193. 10.1111/j.1096-0031.2005.00059.x 34892859

[68] Kück P, Mayer C, Wägele JW, Misof B. Long branch effects distort maximum likelihood phylogenies in simulations despite selection of the correct model. PLoS One. 2012; 7(5): e36593 10.1371/journal.pone.0036593 22662120 PMC3359070

[69] Wang CH, Kuo CH, Mok HK, Lee SC. Molecular phylogeny of elopomorph fishes inferred from mitochondrial 12S ribosomal RNA sequences. Zoologica scripta. 2003; 32(3): 231–41. 10.1046/j.1463-6409.2003.00114.x

[70] Drazen JC, Sutton TT. Dining in the deep: the feeding ecology of deep-sea fishes. Annu Rev Mar Sci. 2017; 9: 337–366. 10.1146/annurev-marine-010816-060543 27814034

[71] Smith KL Jr., Kaufmann RS, Edelman JL, Baldwin RJ. Abyssopelagic fauna in the central North Pacific: comparison of acoustic detection and trawl and baited trap collections to 5800 m. Deep-Sea Res. 1992; 39: 659–685.

[72] Hartel KE, Kenaley CP, Galbraith JK, Sutton TT. Additional records of deep-sea fishes from off greater New England. Northeastern Naturalist. 2008; 15(3): 317–334. 10.1656/1092-6194-15.3.317

[73] Sutton TT, Porteiro FM, Heino M, Byrkjedal I, Langhelle G, Anderson CIH, Horne J, Søiland H, Falkenhaug T, Godø OR, Bergstad OA. Vertical structure, biomass and topographic association of deep-pelagic fishes in relation to a mid-ocean ridge system. Deep-Sea Res II. 2008; 55:161–184.

[74] Castonguay M, McCleave JD. Vertical distributions, diel and ontogenetic vertical migrations and net avoidance of leptocephali of *Anguilla* and other common species in the Sargasso Sea. J Plankton Res. 1987; 9: 195–214.

[75] Smith KF, Brown JH. Patterns of diversity, depth range and body size among pelagic fishes along a gradient of depth. Gobal Ecol Biogeogr. 2002; 11: 313–322.

[76] Bertin L. Sur une series de Leptocephales appartenant au genre *Saccopharynx* mitchill.–C. r. hebd. Séanc. Acad Sci. 1936; Paris 203: 1540–1541.

[77] Bertin L. Formes nouvelles et formes larvaires de poissons Apodes appartenant au sous-ordre des lyomères. Dana Rep. 1938; 15: 1–25.

[78] Bertelsen E, Nielsen JG, Smith DG. Suborder Saccopharyngoidei, families Saccopharyngidae, Eurypharyngidae, and Monognathidae In: Böhlke EB, editor. Fishes of the Western North Atlantic. Sears Foundation for Marine Research, New Haven. Memoir 1 1989; 9(1): 636–655.

[79] Tchernavin VV. Six specimens of Lyomeri in the British Museum (with notes on the skeleton of Lyomeri). J Linn Soc London, Zoology. 1947; 41(279): 287–350.

[80] Tighe KA, Nielsen JG. *Saccopharynx berteli*, a new gulper eel from the Pacific Ocean (Teleostei, Saccopharyngidae). Ichthyol Res. 2000; 47(1): 39–41.

[81] Randall DJ, Farrell AP. Deep-Sea Fishes Academic Press, San Diego; 1997.

[82] Miller MJ, McCleave JD. Species assemblages of leptocephali in the subtropical convergence zone of the Sargasso Sea. J Mar Res. 1994; 52: 743–772.

[83] DeVaney SC. Species distribution modeling of deep pelagic eels. Integr Comp Biol. 2016; 56(4): 524–530. 10.1093/icb/icw032 27252208

[84] Castle PHJ. Notacanthiformes and Anguilliformes: Development In: Moser HG, Richards WJ, editors. Ontogeny and systematics of fishes. American Society of Ichthyologists and Herpetologists. Special Publication 1, Allen Press, Lawrence; 1984: 62–93.

