# Supplementary material for: Resolving deep-sea pelagic saccopharyngiform eel mysteries: Identification of Neocyema and Monognathidae leptocephali and establishment of a new fish family "Neocyematidae" based on larvae, adults and mitogenomic gene orders
Source: PLoS One. 2018 Jul 25;13(7):e0199982. doi: 10.1371/journal.pone.0199982 (PMC6059418; doi:10.1371/journal.pone.0199982)
Supplement: S1 File — (DOCX) [file pone.0199982.s001.docx]

| **Classification** | **Museum** | **Geography** | **Acc. Number** | **Study** |
| --- | --- | --- | --- | --- |
| **Notacanthiformes** |  |  |  |  |
| *Elops saurus* |  |  | AP004807 | Inoue et al. (2004) |
| *Elops hawaiensis* |  |  | AB051070 | Inoue et al. (2004) |
| *Megalops atlanticus* |  |  | AP004808 | Inoue et al. (2004) |
| *Megalops cyprinoides* |  |  | AB051110 | Inoue et al. (2004) |
| *Albula glossodonta* |  |  | AP002973 | Inoue et al. (2004) |
| *Pterothrissus gissu* |  |  | AB051197 | Inoue et al. (2004) |
| *Halosauropsis macrochir* |  |  | AP018130 | Poulsen et al. (2018) |
| *Aldrovandia affinis* |  |  | AP002974 | Inoue et al. (2004) |
| *Aldrovandia oleosa* | ZMUB 21435 | SE Greenland | AP018343 | This study |
| *Notacanthus* cf. *chemniizii* | ZMUB 21950 | SE Greenland | AP018344 | This study |
| *Notacanthus* cf. *chemnitzii* |  |  | AP002975 | Inoue et al. (2003) |
| *Polyacanthonotus rissoanus* | ZMUB 21440 | SE Greenland | AP018342 | This study |
|  |  |  |  |  |
| **Heterenchelyidae** |  |  |  |  |
| *Pythonichthys microphthalmus* |  |  | AP010842 | Inoue et al. (2010) |
|  |  |  |  |  |
| **Myrocongridae** |  |  |  |  |
| *Myroconger compressus* |  |  | AP010847 | Inoue et al. (2010) |
|  |  |  |  |  |
| **Muraenidae** |  |  |  |  |
| *Anarchias sp.* |  |  | AP010843 | Inoue et al. (2010) |
| *Gymnomuraena zebra* |  |  | KP793920 | Loh et al. (2015a) |
| *Rhinomuraena quaesita* |  |  | AP010844 | Inoue et al. (2010) |
| *Gymnothorax formosus* |  |  | KP874184 | Loh et al. (2015c) |
| *Gymnothorax kidako* |  |  | AP002976 | Inoue et al. (2003) |
| *Gymnothorax niphostigmus* | NMMB-P020849 | Ke-Tzu-Liao Taiwan | AP018346 | This study |
| *Scuticaria tigrina* |  |  | KP874183 | Loh et al. (2015c) |
| *Enchelynassa canina* |  |  | KP893074 | Loh et al. (2015b) |
|  |  |  |  |  |
| **Chlopsidae** |  |  |  |  |
| *Kaupichthys hyoproroides* |  |  | AP010845 | Inoue et al. (2010) |
| *Robinsia catherinae* |  |  | AP010846 | Inoue et al. (2010) |
|  |  |  |  |  |
| **Derichthyidae** |  |  |  |  |
| *Nessorhamphus ingolfianus* |  |  | AP010850 | Inoue et al. (2010) |
| *Derichthys serpentinus* |  |  | AP010851 | Inoue et al. (2010) |
|  |  |  |  |  |
| **Colocongridae** |  |  |  |  |
| *Coloconger cadenati* |  |  | AP010863 | Inoue et al. (2010) |
| *Thalassenchelys sp.* |  |  | AP010867 | Inoue et al. (2010) |
|  |  |  |  |  |
| **Congridae** |  |  |  |  |
| *Ariosoma meeki* |  |  | KX641476 | Xu (unpubl.) |
| *Ariosoma shiroanago* |  |  | AP010861 | Inoue et al. (2010) |
| *Heteroconger hassi* |  |  | AP010859 | Inoue et al. (2010) |
| *Paraconger notialis* |  |  | AP010860 | Inoue et al. (2010) |
| *Conger japonicus* |  |  | KR131863 | Xu & Wei (Unpubl.) |
| *Conger myriaster* |  |  | AB038381 | Inoue et al. (2001a) |
|  |  |  |  |  |
| **Ophichthidae** |  |  |  |  |
| *Myrichthys maculosus* |  |  | AP010862 | Inoue et al. (2010) |
| *Ophisurus macrorhynchos* |  |  | AP002978 | Inoue et al. (2004) |
|  |  |  |  |  |
| **Muraenesocidae** |  |  |  |  |
| *Muraenesox bagio* |  |  | AP010852 | Inoue et al. (2010) |
| *Cynoponticus ferox* |  |  | AP010853 | Inoue et al. (2010) |
|  |  |  |  |  |
| **Nettastomatidae** |  |  |  |  |
| *Hoplunnis punctata* |  |  | AP010865 | Inoue et al. (2010) |
| *Facciolella oxyrhyncha* |  |  | AP010866 | Inoue et al. (2010) |
| *Leptocephalus* sp. (larvae) |  |  | AP010868 | Inoue et al. (2010) |
| *Nettastoma parviceps* |  |  | AP010864 | Inoue et al. (2010) |
|  |  |  |  |  |
| **Protoanguillidae** |  |  |  |  |
| *Protoanguilla palau* |  |  | AP011809 | Johnson et al. (2012) |
|  |  |  |  |  |
| **Synaphobranchidae** |  |  |  |  |
| *Ilyophis brunneus* |  |  | AP010848 | Inoue et al. (2010) |
| *Synaphobranchus kaupii* |  |  | AP002977 | Inoue et al. (2004) |
| *Simenchelys parasitica* |  |  | AP010849 | Inoue et al. (2004) |
|  |  |  |  |  |
| **Serrivomeridae** |  |  |  |  |
| *Serrivomer sector* |  |  | AP007250 | Minegishi et al. (2005) |
| *Serrivomer beanii* |  |  | AP010857 | Inoue et al. (2010) |
| *Stemonidium hypomelas* |  |  | AP010858 | Inoue et al. (2010) |
|  |  |  |  |  |
| **Nemichthyidae** |  |  |  |  |
| *Nemichthyidae* |  |  | AP010856 | Inoue et al. (2010) |
| *Avocettina infans* |  |  | AP010855 | Inoue et al. (2010) |
| *Nemichthys scolopaceus* |  |  | AP010854 | Inoue et al. (2010) |
|  |  |  |  |  |
| **Anguillidae** |  |  |  |  |
| *Anguilla malgumora* |  |  | AP007238 | Minegishi et al. (2005) |
| *Anguilla rostrata* |  |  | AP007249 | Minegishi et al. (2005) |
| *Anguilla anguilla* |  |  | AP007233 | Minegishi et al. (2005) |
| *Anguilla dieffenbachi* |  |  | AP007240 | Minegishi et al. (2005) |
| *Anguilla australis australis* |  |  | AP007234 | Minegishi et al. (2005) |
| *Anguilla australis schmidti* |  |  | AP007235 | Minegishi et al. (2005) |
| *Anguilla mossambica* |  |  | AP007244 | Minegishi et al. (2005) |
| *Anguilla japonica* |  |  | AB038556 | Inoue et al. (2001b) |
| *Anguilla megastoma* |  |  | AP007243 | Minegishi et al. (2005) |
| *Anguilla celebesensis* |  |  | AP007239 | Minegishi et al. (2005) |
| *Anguilla bicolor bicolor* |  |  | AP007236 | Minegishi et al. (2005) |
| *Anguilla bicolor pacifica* |  |  | AP007237 | Minegishi et al. (2005) |
| *Anguilla obscura* |  |  | AP007247 | Minegishi et al. (2005) |
| *Anguilla bengalensis bengalensis* |  |  | KT895265 | Mohindra et al. (2015) |
| *Anguilla bengalensis labiata* |  |  | AP007245 | Minegishi et al. (2005) |
| *Anguilla bengalensis nebulosa* |  |  | AP007246 | Minegishi et al. (2005) |
| *Anguilla luzonensis* |  |  | AB469437 | Watanabe et al. (2009) |
| *Anguilla interioris* |  |  | AP007241 | Minegishi et al. (2005) |
| *Anguilla marmorata* |  |  | AP007242 | Minegishi et al. (2005) |
| *Anguilla reinhardtii* |  |  | AP007248 | Minegishi et al. (2005) |
|  |  |  |  |  |
| **Moringuidae** |  |  |  |  |
| *Moringua edwardsi* |  |  | AP010840 | Inoue et al. (2010) |
| *Moringua microchir* |  |  | AP010841 | Inoue et al. (2010) |
|  |  |  |  |  |
| **Neocyematidae** |  |  |  |  |
| *Neocyema erythrosoma* | ZMUB 21865 | SE Greenland | AP018345 | This study |
|  |  |  |  |  |
| **Monognathidae** |  |  |  |  |
| *Monognathus jesperseni* |  |  | AP010869 | Inoue et al. (2010) |
|  |  |  |  |  |
| **Cyematidae** |  |  |  |  |
| *Cyema atrum* |  |  | AP010870 | Inoue et al. (2010) |
|  |  |  |  |  |
| **Saccopharyngidae** |  |  |  |  |
| *Saccopharynx lavenbergi* |  |  | AB047825 | Inoue et al. (2003) |
|  |  |  |  |  |
| **Eurypharyngidae** |  |  |  |  |
| *Eurypharynx pelecanoides* |  |  | AB046473 | Inoue et al. (2003) |

**References for mitogenomic data**

Loh KH, Shao KT, Chen HM, Chen CH, Chong VC, Loo PL, Shen KN, Hsiao CD. Next generation sequencing yields the complete mitochondrial genome of the Zebra moray, *Gymnomuraena zebra* (Anguilliformes: Muraenidae). Mitochondrial DNA Part A. 2015a; 27(6): 4230–4231. doi: [10.3109/19401736.2015.1022754](http://dx.doi.org/10.3109/19401736.2015.1022754)

Loh KH, Shao KT, Chen HM, Chen CH, Loo PL, Hui ATY, Lim PE, Chong VC, Shen KN, Hsiao CD. Next-generation sequencing yields the complete mitochondrial genome of the longfang moray, *Enchelynassa canina* (Anguilliformes: Muraenidae). Mitochondrial DNA Part A. 2015b; 27(4): 2431–2432. doi: [10.3109/19401736.2015.1030629](http://dx.doi.org/10.3109/19401736.2015.1030629)

Loh KH, Shao KT, Chen CH, Chen HM, Then AYH, Loo PL, Lim PE, Chong VC, Shen KN, Hsiao CD. Complete mitogenome of two moray eels of *Gymnothorax formosus* and *Scuticaria tigrina* (Anguilliformes: Muraenidae). Mitochondrial DNA Part A. 2015c; 27(4). doi: [10.3109/19401736.2015.1043530](http://dx.doi.org/10.3109/19401736.2015.1043530)

Inoue JG, Miya M, Tsukamoto K, Nishida M. Complete mitochondrial DNA sequence of *Conger myriaster* (Teleostei: Anguilliformes): novel gene order for vertebrate mitochondrial genomes and the phylogenetic implications for anguilliform families. J Mol Evol. 2001a; 52(4): 311–320.

Inoue JG, Miya M, Aoyama J, Ishikawa S, Tsukamoto K, Nishida M. Complete mitochondrial DNA sequence of the Japanese eel *Anguilla japonica*. Fish Sci*.* 2001b; 67(1): 118–125.

Inoue JG, Miya M, Tsukamoto K, Nishida M. Evolution of the deep-sea gulper eel mitochondrial genomes: large-scale gene rearrangements originated within the eels. Mol Biol Evol. 2003. 20(11): 1917–24. doi: [10.1093/molbev/msg206](https://doi.org/10.1093/molbev/msg206)

Inoue JG, Miya M, Tsukamoto K, Nishida M. Mitogenomic evidence for the monophyly of elopomorph fishes (Teleostei) and the evolutionary origin of the leptocephalus larva. Mol Phylogenet Evol. 2004; 32: 274–286. doi:10.1016/j.ympev.2003.11.009

Inoue JG, Miya M, Miller MJ, Sado T, Hanel R, Hatooka K, Aoyama J, Minegishi Y, Nishida M, Tsukamoto K. Deep-ocean origin of the freshwater eels. Biol Lett. 2010; 6: 363–366. doi:10.1098/rsbl.2009.0989

Johnson GD, Ida H, Sakaue J, Sado T, Asahida T, Miya M. A `living fossil`eel (Anguilliformes: Protoanguillidae, fam. nov.) from an undersea cave in Palau. Proc R. Soc B. 2012; 279(1730). doi: 10.1098/rspb.2011.1289

Minegishi Y, Aoyama J, Inoue JG, Miya M, Nishida M, Tsukamoto K. Molecular phylogeny and evolution of the freshwater eels genus *Anguilla* based on the whole mitochondrial genome sequences. Mol Phylogenet Evol. 2005; 34(1): 134–146.

Mohindra V, Singh RK, Tripathi RK, Lal KK, Jena JK. Complete mitogenome of Indian mottled eel, *Anguilla bengalensis bengalensis* (Gray, 1831) through PacBio RSII sequencing. Mitochondrial DNA. 2015; 1–2.

Poulsen JY, Thorkildsen S, Hammeken NA. Identification keys to halosaurs and notacanthids (Notacanthiformes, Elopomorpha) in the subarctic North Atlantic Ocean including three new species records and multiple molecular OTUs of *Notacanthus* cf. *chemnitzii*. Mar Biodiv. 2018; 48(2): 1009–1025. doi[: 10.1007/s12526-017-0762-8](http://dx.doi.org/10.1007/s12526-017-0762-8)

Watanabe S, Aoyama J, Tsukamoto K. A new species of freshwater eel *Anguilla luzonensis* (Teleostei: Anguillidae) from Luzon Island of the Philippines. Fish Sci. 2009; 75(2): 387–392.
