# Supplementary figures and images for: Resolving deep-sea pelagic saccopharyngiform eel mysteries: Identification of Neocyema and Monognathidae leptocephali and establishment of a new fish family "Neocyematidae" based on larvae, adults and mitogenomic gene orders
Source: PLoS One. 2018 Jul 25;13(7):e0199982. doi: 10.1371/journal.pone.0199982 (PMC6059418; doi:10.1371/journal.pone.0199982)

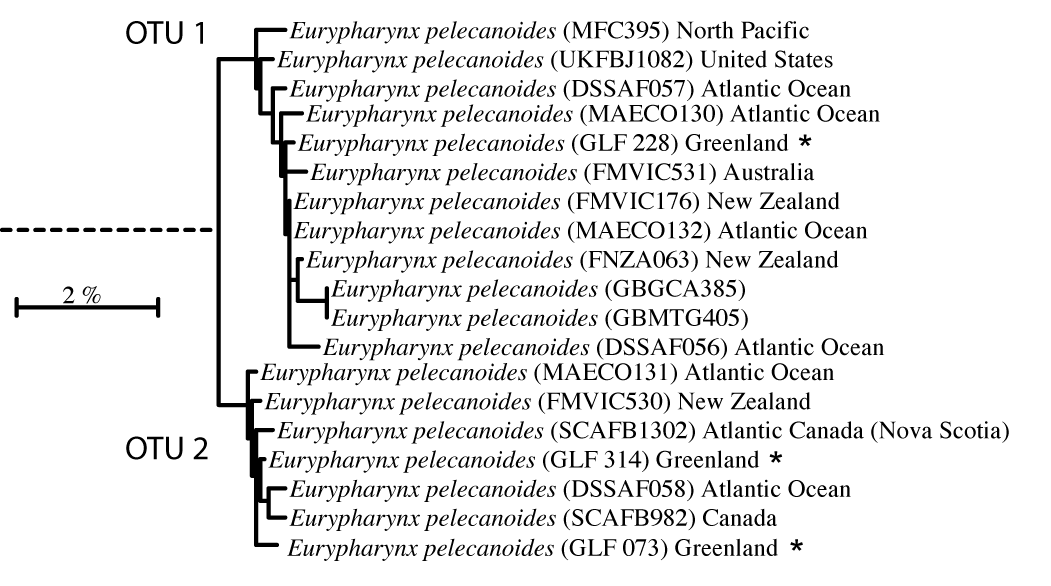

Supplement: S4 File — Materials and methods are presented in Poulsen et al. [60] and Greenland records (GLF records) can be found in the BOLD repository under the Greenland Fishes Barcoding Project (Poulsen et al.) [60]. Note how the two OTUs of Eurypharynx cf. pelecanoides show no associations with geography of samples. (TIF) [file pone.0199982.s004.tif]
